# Modelling and verification of reconfigurable multi-agent systems

**DOI:** 10.1007/s10458-021-09521-x

**Published:** 2021-08-26

**Authors:** Yehia Abd Alrahman, Nir Piterman

**Affiliations:** University of Gothenburg, Gothenburg, Sweden

**Keywords:** Agent theories and models, Logics for agent reasoning, Verification of multi-agent systems

## Abstract

We propose a formalism to model and reason about reconfigurable multi-agent systems. In our formalism, agents interact and communicate in different modes so that they can pursue joint tasks; agents may dynamically synchronize, exchange data, adapt their behaviour, and reconfigure their communication interfaces. Inspired by existing multi-robot systems, we represent a system as a set of agents (each with local state), executing independently and only influence each other by means of message exchange. Agents are able to sense their local states and partially their surroundings. We extend ltl to be able to reason explicitly about the intentions of agents in the interaction and their communication protocols. We also study the complexity of satisfiability and model-checking of this extension.

## Introduction

In recent years formal modelling of multi-agent systems (MAS) and their analysis through model checking has received much attention [42, 61]. Several mathematical formalisms have been suggested to represent the behaviour of such systems and to reason about the strategies that agents exhibit [9, 42]. For instance, modelling languages, such as RM [8, 33] and ISPL [42], are used to enable efficient analysis by representing these systems through the usage of BDDs. Temporal logics have been also extended and adapted (e.g., with knowledge and epistemic operators [27, 31]) specifically to support multi-agent modelling [32]. Similarly, logics that support reasoning about the intentions and strategic abilities of such agents have been used and extended [22, 50].

These works are heavily influenced by the formalisms used for verification (e.g., Reactive Modules [6, 8], concurrent game structures [9], and interpreted systems [42]). They rely on shared memory to implicitly model interactions. It is generally agreed that explicit message passing is more appropriate to model interactions among distributed agents because of its scalability [13, 37]. However, the mentioned formalisms trade the advantages of message passing for abstraction, and abstract message exchange by controlling the visibility of state variables of the different agents.

Furthermore, the compositionality of shared memory approaches is limited and the supported interaction interfaces are in general not very flexible [14]. Alternatively, message passing formalisms [48] are very compositional and support flexible interaction interfaces. However, unlike shared memory formalisms, they do not accurately support awareness capabilities, where an agent may instantaneously inspect its local state and adapt its behaviour while interacting. The reason is that they model agents as mathematical expressions over interaction operators. Thus the state of an agent is implicit in the structure of the expression.

Based on an early result, where a translation from shared memory to message passing was provided [11], it was believed that a shared memory model is a higher level abstraction of distributed systems. However, this result holds only in specific cases and under assumptions that practically proved to be unrealistic. As discussed in [3], the translation was not provided in a standard way where every feature of one formalism is efficiently translated to a corresponding one in the other formalism, but rather based on emulation. That is, it computationally shows if a problem has a solution in one formalism it also has one in the other formalism. However, this is not surprising as most computational formalisms are Turing powerful. A good translation (See [2]) should also preserve the observable behaviour of the translated process and its divergence tendencies. This is important in distributed settings because the observable behaviour of a process defines its communication capabilities, which can be influenced in open world settings. Thus the above mentioned translation only works under closed world assumption and does not capture divergence. Namely, a timely process in one formalism may diverge indefinitely while awaiting for other processes. Furthermore, the translation cannot deal with failure or anonymous interaction, and thus requires that there is a majority of correct processes and a pre-defined knowledge of each other’s identities and the number of processes in the systems. Thus, there is no way to model the birth/death of processes in the system.

To combine the benefits of both approaches recent developments [3, 59] suggest adopting hybrids, that accurately represent actual distributed systems, e.g.,  [4, 46]. We propose a hybrid formalism to model and reason about distributed multi-agent systems. A system is represented as a set of agents (each with local state), executing concurrently and only interacting by message exchange. Inspired by multi-robot systems, e.g., Kilobot [52] and Swarmanoid [23], agents are additionally able to sense their local states and partially their surroundings. Interaction is driven by message passing following the interleaving semantics of [48]; in that only one agent may send a message at a time while other agents may react to it. To support meaningful interaction among agents [60], messages are not mere synchronisations, but carry data that might be used to influence the behaviour of receivers.

Our message exchange is adaptable and reconfigurable. Thus, agents determine how to communicate and with whom. Agents interact on links that change their utility based on the needs of interaction at a given stage. Unlike existing message-passing mechanisms, which use static notions of network connectivity to establish interactions, our mechanisms allow agents to specify receivers using logical formulas. These formulas are interpreted over the evolving local states of the different agents and thus provide a natural way to establish reconfigurable interaction interfaces (for example, limited range communication [46], messages destined for particular agents [2], etc.).

The advantages of our formalism are threefold. We provide more realistic models that are close to their distributed implementations, and how actual distributed MAS are developed, e.g., [36]. We provide a modelling convenience for high level interaction features of MAS (e.g., coalition formation, collaboration, self-organisation, etc), that otherwise have to be hard-coded tediously in existing formalisms. Furthermore, we decouple the individual behaviour of agents from their interaction protocols to facilitate reasoning about either one separately.

In addition, we extend ltl to characterise messages and their targets. This way we allow reasoning about the intentions of agents in communication. Our logic can refer directly to the interaction protocols. Thus the interpretation of a formula incorporates information about the causes of assignments to variables and the flow of the interaction protocol. We also study the complexity of satisfiability and Model-checking for our logic.

This article is an extended and revised version of the conference paper presented in [1]. The major extensions in this article consist of: (i) a compositional and enumerative semantic definition of the proposed formalism, that coincides with the early symbolic one. The new definition facilitates reasoning about the individual behaviour of agents and their compositions with others. For this purpose, we defined a parallel composition operator with reconfigurable broadcast and multicast semantics. Thus, the definition is not only intuitive, but can also be used to reason about models under *open-world assumption*; (ii) a major improvement on our early results [1] regarding satisfiability and model checking, that were computed in an expspace upper bound. Here, we provide a novel automata construction that permits pspace analysis, matching the lower bound. Thus, this part is majorly rewritten and improved. Moreover, we enhance the presentation of the different parts of the article and provide the proofs of all results.

The structure of this article is as follows: In Sect. 2, we informally present our formalism and motivate our design choices. In Sect. 3, we give the necessary background and in Sect. 4 we present the compositional semantic definition. In Sect. 5 we introduce the formalism both in terms of enumerative and symbolic semantics, and we prove that they coincide. In Sect. 6, we present a non-trivial case study to show the distinctive features of our formalism. In Sect. 7 we discuss our extension to LTL and provide efficient decision procedures to check both satisfiability and model checking in polynomial space. Finally, in Sect. 8 we report closely related works and in Sect. 9 we discuss our concluding remarks.

## An informal overview

We use a collaborative-robot scenario to informally illustrate the distinctive features of our formalism and we later formalise it in Sect. 6. The scenario is based on Reconfigurable Manufacturing Systems (RMS) [38, 44], where assembly product lines coordinate autonomously with different types of robots to produce products.

In our formalism, each agent has a local state consisting of a set of variables whose values may change due to either contextual conditions or side-effects of interaction. The external behaviour of an agent is only represented by the messages it exposes to other agents while the local one is represented by changes to its state variables. These variables are initialised by initial conditions and updated by send- and receive- transition relations. In our example, a product-line agent initiates different production procedures based on the assignment to its product variable $``{\mathsf {prd}}"$, which is set by the operator, while it controls the progress of its status variable $``{\mathsf {st}}"$ based on interactions with other robots. Furthermore, a product-line agent is characterised: (1) externally only by the recruitment and assembly messages it sends to other robots and (2) internally by a sequence of assignments to its local variables.

Before we explain the send- and receive- transition relations and show the dynamic reconfiguration of communication interfaces we need to introduce a few additional features. We assume that there is an agreed set of *channels/links* ${\textsc {ch}}$ that includes a unique broadcast channel $\star$. Broadcasts have non-blocking send and blocking receive while multicasts have blocking send and receive. In a broadcast, receivers (if exist) may anonymously receive a message when they are interested in its values and when they satisfy the send guard. Otherwise, the agent does not participate in the interaction either because they cannot (do not satisfy the guard) or because they are not interested (make an idle transition). In multicast, all agents connected to the multicast channel must participate to enable the interaction. For instance, recruitment messages are broadcast because a line agent assumes that there exist enough robots to join the team while assembly messages are multicast because they require that the whole connected team is ready to assemble the product.

Agents dynamically decide (based on local state) whether they can use (i.e., connect-to) multicast channels while the broadcast channel is always available. Thus, initially, agents may not be connected to any channel, except for the broadcast one ${\star }$. These channels may be learned using broadcast messages and thus a structured communication interface can be built at run-time, starting from a (possibly) flat one.

Agents use messages to send selected data and specify how and to whom. Namely, the values in a message specify what is exposed to the others; the channel specifies how to coordinate with others; and a send guard specifies the target. Accordingly, each message carries an assignment to a set of agreed *data variables* ${\textsc {d}}$, i.e., the exposed data; a channel $\mathsf {ch}$; and a send guard ${g}^{s}_{}$. In order to write meaningful send guards, we assume a set of *common variable names* ${\textsc {cv}}$ (*common variables*, for short). Each agent has local variables that are identified by these names. Agents assign their own individual information to these local variables (e.g., the type of agent, its location, its readiness, etc.). Send guards are expressed in terms of conditions on these names and are evaluated per agent based on their assigned local values. Send guards are parametric to the local state of the sender and specify what assignments to the common variables a potential receiver must have. For example, an agent may send a dedicated link name to a selected set of agents by assigning a data variable in the communicated message and this way a coalition can be built incrementally at run-time. In our RMS, the send guard of the recruitment message specifies the types of the targeted robots while the data values expose the number of required robots per type and a dedicated multicast link to be used to coordinate the production.

Targeted agents may use incoming messages to update their states, reconfigure their interfaces, and/or adapt their behaviour. In order to do so, however, agents are equipped with receive guards ${g}^{r}_{}$; that might be parametrised to local variables and channels, and thus dynamically determine if an agent is connected to a given channel. The interaction among different agents is then derived based on send- and receive- transition relations. These relations are used to decide when to send/receive a message and what are the side-effects of interaction. Technically, every agent has a send and a receive transition relation. Both relations are parameterised by the state variables of the agent, the data variables transmitted on the message, and by the channel name. A sent message is interpreted as a joint transition between the send transition relation of the sender and the receive transition relations of all the receivers. For instance, a robot’s receive guard specifies that other than the broadcast link it is also connected to a multicast link that matches the current value of its local variable $``{\mathsf {lnk}}"$. The robot then uses its receive transition relation to react to a recruitment message, for instance, by assigning to its $``{\mathsf {lnk}}"$ the link’s data value from the message.

Furthermore, in order to send a message the following has to happen. The send transition relation of the sender must hold on: a given state of the sender, a channel name, and an assignment to data variables. If the message is broadcast, all agents whose assignments to common variables satisfy the send guard jointly receive the message, the others discard it. If the message is multicast, all connected agents must satisfy the send guard to enable the transmission (as otherwise they block the message). In both cases, sender and receivers execute their send- and receive-transition relations jointly. The local side-effect of the message takes into account the origin local state, the channel, and the data. In our example, a (broadcast) recruitment message is received by all robots that are not assigned to other teams (assigned ones discard it) and as a side effect they connect to a multicast channel that is specified in the message. A (multicast) assembly message can only be sent when the whole recruited team is ready to receive (otherwise the message is blocked) and as a side effect the team proceeds to the next production stage.

Clearly, the dynamicity of our formalism stems from the fact that we base interactions directly over the evolving states of the different agents rather than over static notions of network connectivity as of existing approaches.

## Transition systems and finite automata

We unify notations and give the necessary background. We introduce doubly-labeled transition systems and discrete systems and show how to translate the former to the latter. We further introduce nondeterministic and alternating Büchi word automata.

### Transition systems and discrete systems

A *Doubly-Labeled Transition System* (TS) is $\mathcal{T}=\langle \varSigma ,\varUpsilon ,S,S_0,R,L\rangle$, where $\varSigma$ is a *state alphabet*, $\varUpsilon$ is a *transition alphabet*, *S* is a set of states, $S_0\subseteq S$ is a set of initial states, $R\subseteq S\times \varUpsilon \times S$ is a transition relation, and $L:S\rightarrow \varSigma$ is a labeling function.

A *path* of a transition system $\mathcal{T}$ is a maximal sequence of states and transition labels $\sigma =s_0,a_0,s_1,a_1,\ldots$ such that $s_0\in S_0$ and for every $j\ge 0$ we have $(s_i,a_i,s_{i+1})\in R$. We assume that for every state $s\in S$ there are $a\in \varUpsilon$ and $s'\in S$ such that $(s,a,s')\in R$. Thus, a sequence $\sigma$ is maximal if it is infinite. If $|\varUpsilon |=1$ then $\mathcal{T}$ is a *state-labeled transition system* and if $|\varSigma |=1$ then $\mathcal{T}$ is a *transition-labeled transition system*.

We introduce *Discrete Systems* (DS) that represent state-labeled systems symbolically. A DS is $\mathscr {D} = \langle \mathscr {V}, \theta , \rho \rangle$, where the components of $\mathscr {D}$ are as follows:

- $\mathscr {V} = \{{v_1,\ldots ,v_n}\}$: A finite set of typed variables. Variables range over discrete domains, e.g., Boolean or Integer. A *state* *s* is an interpretation of $\mathscr {V}$, i.e., if $D_v$ is the domain of *v*, then *s* is in $\prod _{v_i\in \mathscr {V}} D_{v_i}$.
  We assume some underlying first-order language over $\mathscr {V}$ that includes (i) *expressions* constructed from the variables in $\mathscr {V}$, (ii) *atomic formulas* that are either Boolean variables or the application of different predicates to expressions, and (iii) *assertions* that are first-order formulas constructed from atomic formulas using Boolean connectives or quantification of variables. Assertions, also sometimes called *state formulas*, characterize states through restriction of possible variable values in them.
- $\theta$ : This is an assertion over $\mathscr {V}$ characterising all the initial states of the DS. A state is called *initial* if it satisfies $\theta$.
- $\rho$ : A *transition relation*. This is an assertion $\rho (\mathscr {V}\cup \mathscr {V}')$, where $\mathscr {V}'$ is a primed copy of variables in $\mathscr {V}$. The transition relation $\rho$ relates a state $s\in \varSigma$ to its $\mathscr {D}$-*successors* $s'\in \varSigma$, i.e., $(s,s')\models \rho$, where *s* is an interpretation to variables in $\mathscr {V}$ and $s'$ is for variables in $\mathscr {V}'$.

The DS ${\mathscr {D}}$ gives rise to a state transition system $\mathscr {T}_{\mathscr {D}}=\langle \varSigma ,\{1\},T,T_0,R\rangle$, where $\varSigma$ and *T* are the set of states of $\mathscr {T}_\mathscr {D}$, $T_0$ is the set of initial states, and *R* is the set of triplets $(s,1,s')$ such that $(s,s')\models \rho$. Clearly, the paths of ${\mathscr {T}}_{\mathscr {D}}$ are exactly the paths of $\mathscr {D}$, but the size of $\mathscr {T}_\mathscr {D}$ is exponentially larger than the description of $\mathscr {D}$.

A common way to translate a DLTS into a DS, which we adapt and extend below, would be to include additional variables that encode the transition alphabet. Given such a set of variables $\mathscr {V}_\varUpsilon$, an assertion $\rho (\mathscr {V} \cup \mathscr {V}_\varUpsilon \cup \mathscr {V}')$ characterises the triplets $(s,\upsilon ,s')$ such that $(s,\upsilon ,s') \models \rho$, where *s* supplies the interpretation to $\mathscr {V}$, $\upsilon$ to $\mathscr {V}_\varUpsilon$ and $s'$ to $\mathscr {V}'$.

### Finite automata on infinite words

We use the automata-theoretic approach to linear temporal logic [58]. Thus, we translate temporal logic formulas to automata. We give here the necessary background.

For an alphabet $\varSigma$, the set $\varSigma ^{\omega }$ is the set of infinite sequences of elements from $\varSigma$. Given an alphabet $\varSigma$ and a set *D* of directions, a $\varSigma$-*labeled* *D*-*tree* is a pair $\left( T,\tau \right)$, where $T \subseteq D^*$ is a tree over *D* and $\tau :T \rightarrow \varSigma$ maps each node of *T* to a letter in $\varSigma$. A *path* $\pi$ of a tree *T* is a set $\pi \subseteq T$ such that $\epsilon \in \pi$ and for every $x\in \pi$ either *x* is a leaf in *T* or there exists a unique $\gamma \in D$ such that $x\cdot \gamma \in \pi$. For $\pi =\gamma _1\cdot \gamma _2\cdots$, we write $\tau (\pi )$ for $\tau (\epsilon )\cdot \tau (\gamma _1)\cdot \tau (\gamma _1\gamma _2) \cdots$.

For a finite set *X*, let  be the set of positive Boolean formulas over *X* (i.e., Boolean formulas built from elements in *X* using $\wedge$ and $\vee$), where we also allow the formulas $\mathsf{true}$ and $\mathsf{false}$. For a set $Y\subseteq X$ and a formula , we say that *Y* *satisfies* $\theta$ iff assigning $\mathsf{true}$ to elements in *Y* and assigning $\mathsf{false}$ to elements in $X{\setminus } Y$ makes $\theta$ true.

#### Definition 1

*(Alternating Büchi Word Automata (ABW))* An alternating Büchi word automaton is of the form $A=\langle \varSigma$, *Q*, $q_{in}$, $\delta$, $F\rangle$, where $\varSigma$ is the input alphabet, *Q* is a finite set of states,  is a transition function, $q_{in} \in Q$ is an initial state, and $F \subseteq Q$ specifies a Büchi acceptance condition.

A run of an ABW *A* on $w=\sigma _0\sigma _1\ldots$ is a *Q*-labeled *D*-tree, $\left( T,\tau \right)$, where $\tau (\epsilon )=q_{in}$ and, for every $x\in T$, we have $\{\tau (x\cdot \gamma _1),\ldots , \tau (x\cdot \gamma _k)\} \models \delta (\tau (x),\sigma _{|x|})$ where $\{x\cdot \gamma _1,\ldots , x\cdot \gamma _k\}$ is the set of children of *x*. A run of *A* is accepting if all its infinite paths satisfy the acceptance condition. For a path $\pi$, let $inf(\pi )=\{q ~|~ q \hbox { appears infinitely often in }\tau (\pi )\}$. A path $\pi$ is accepting if $inf(\pi ) \cap F \ne \emptyset$. Thus, every infinite path in the run tree must visit the acceptance set *F* infinitely often. The ABW *A* accepts *w* if there exists an accepting run on *w*. We denote by $L_{\omega }({A})$ the set of words accepted by *A*.

#### Definition 2

*(Nondeterministic Büchi Word Automata (NBW))* A NBW is $N=\langle \varSigma$, *Q*, $Q_{in}$, $\delta$, $F\rangle$, where $\varSigma$ is an input alphabet, *Q* is a finite set of states, $\delta :Q \times \varSigma \rightarrow 2^{Q}$ is a transition function, $Q_{in} \subseteq Q$ is a set of initial state, and $F \subseteq Q$ specifies a Büchi acceptance condition.

A run of a NBW *N* on $w=\sigma _0\sigma _1\ldots \in \varSigma ^{\omega }$ is a sequence $r=q_0q_1\ldots \in Q^{\omega }$ such that $q_0\in Q_{in}$, and for all $i\ge 0$ we have $q_{i+1}\in \delta ({q_i,\sigma _{i+1}})$. A run is *accepting* if $inf(r) \cap F \ne \emptyset$. The NBW *N* accepts *w* if there exists an accepting run of *N* on *w*. We denote by $L_{\omega }({N})$ the set of words accepted by *N*.

We state the following well known results about Linear Temporal Logic (LTL), NBW, and ABW (omitting the definition of LTL).

#### Theorem 1

([57, 58]) For every LTL formula $\varphi$ of length *n* there exist an ABW $A_\varphi$ with *O*(*n*) states such that $L(A_\varphi )=L(\varphi )$.

#### Theorem 2

([49]) For every ABW *A* with *n* states there is an NBW *N* such that $L_{\omega }({N})=L_{\omega }({A})$. The number of states of *N* is in $2^{\mathscr {O}({n})}$.

## Channelled transition systems

In this section, we propose *Channelled Transition System (CTS)* to facilitate compositional modelling of interactive systems. Namely, we extend the format of transition labels of Doubly-Labelled Transition Systems to also specify the role of the transition (i.e., send- or receive- message) and the used communication channels. We define a parallel composition operator that considers both broadcast and multicast semantics and we study its properties. The techniques to prove these results are rather standard. However, we are not familiar with a setup that conveniently allows the existence of transitions to depend on subscription to channels as we suggest below.

### Channelled transition systems (CTS)

A *Channelled Transition System* (CTS) is $\mathscr {T}=\langle C, \varSigma ,\varUpsilon ,S, S_0,R,L,{\textsc {ls}}\rangle$, where *C* is a set of channels, including the broadcast channel ($\star$), $\varSigma$ is a *state alphabet*, $\varUpsilon$ is a *transition alphabet*, *S* is a set of states, $S_0\subseteq S$ is a set of initial states, $R\subseteq S\times \varUpsilon \times S$ is a transition relation, $L:S\rightarrow \varSigma$ is a labelling function, and ${\textsc {ls}}:S \rightarrow 2^C$ is a channel-listening function such that for every $s\in S$ we have $\star \in {\textsc {ls}}(s)$. We assume that $\varUpsilon = \varUpsilon ^+ \times \{!,?\} \times C$, for some set $\varUpsilon ^+$. That is, every transition labelled with some $\upsilon \in \varUpsilon ^+$ is either a message send (!) or a message receive (?) on some channel $c\in C$.

A *path* of a CTS $\mathscr {T}$ is a maximal sequence of states and transition labels $\sigma =s_0,a_0,s_1,a_1,\ldots$ such that $s_0\in S_0$ and for every $i\ge 0$ we have $(s_i,a_i,s_{i+1})\in R$. As before, we assume that for every state $s\in S$ there exist $a\in \varUpsilon$ and $s'\in S$ such that $(s,a,s')\in R$. Thus, a sequence $\sigma$ is maximal if it is infinite.

#### Remark 1

Note that the transition labels $a_i$ of a CTS’s path $\sigma =s_0,a_0,s_1,a_1,\ldots$ range over both send (!) and receive (?) transitions. Depending on the underlying semantics of the CTS, send transitions may happen independently regardless of the existence of receivers, e.g., in case of broadcast semantics. However, receive transitions may only happen jointly with some send transition. By allowing CTS’s paths to also range over receive transitions, we can model every system as a collection of (open) systems that interact through message exchange. That is, a receive transition in a system is a hole that is closed/filled when composed with a send transition from another system. A complete system (i.e., with filled holes) is called a *closed system*.

The analysis in this article considers closed systems where a system path ranges over send transitions only. In other words, we only consider the messages exchanged within the system under consideration.

The parallel composition of systems is defined below.

#### Definition 3

*(Parallel Composition)* Given two CTS $\mathscr {T}_i=\langle C_i, \varSigma _i,\varUpsilon _i,S_i, S^i_0,R_i,L_i,{\textsc {ls}}^i\rangle$, where $i\in \{1,2\}$ their composition $\mathscr {T}_1\parallel \mathscr {T}_2$ is the following CTS $\mathscr {T}=\langle C, \varSigma ,\varUpsilon ,S, S_0,R,L,{\textsc {ls}}\rangle$, where the components of $\mathscr {T}$ are:

- $$C = C_1 \cup C_2$$
- $$\varSigma = \varSigma _1 \times \varSigma _2$$
- $$\varUpsilon = \varUpsilon ^1 \cup \varUpsilon ^2$$
- $$S = S_1\times S_2$$
- $$S_0 = S_0^1 \times S_0^2$$
- $$R = $$
- $$L(s_1,s_2) = (L_1(s_1),L_2(s_2))$$
- $${\textsc {ls}}(s_1,s_2) = {\textsc {ls}}^1(s_1)\cup {\textsc {ls}}^2(s_2)$$

The transition relation *R* of the composition defines two modes of interactions, namely multicast and broadcast. In both interaction modes, the composition $\mathscr {T}$ sends a message $(\upsilon ,!,c)$ on channel *c* (i.e., $((s_1,s_2),(\upsilon ,!,c),(s'_1,s'_2))\in R$) if either $\mathscr {T}_1$ or $\mathscr {T}_2$ is able to generate this message, i.e, $(s_1,(\upsilon ,!,c),s'_1)\in R_1$ or $(s_2,(\upsilon ,!,c),s'_2)\in R_2$.

Consider the case of a multicast channel. A multicast is blocking. Thus, a multicast message is sent if either it is received or the channel it is sent on is not listened to. Suppose that a message originates from $\mathscr {T}_1$, i.e., $(s_1,(\upsilon ,!,c),s'_1)\in R_1$. Then, $\mathscr {T}_2$ must be able to either receive the message or, in the case that $\mathscr {T}_2$ does not listen to the channel, discard it. CTS $\mathscr {T}_2$ receives if $(s_2,(\upsilon ,?,c),s'_2)\in R_2$. It discards if $c\notin {\textsc {ls}}^2(s_2)$ and $s_2=s'_2$. The case of $\mathscr {T}_2$ sending is dual. Note that $\mathscr {T}_2$ might be a composition of other CTS(s), say $\mathscr {T}_2=\mathscr {T}_3\Vert \mathscr {T}_4$. In this case, $\mathscr {T}_2$ listens to channel *c* if at least one of $\mathscr {T}_3$ or $\mathscr {T}_4$ is listening. That is, it could be that either $c\in ({\textsc {ls}}(s_3)\cap {\textsc {ls}}(s_4))$, $c\in ({\textsc {ls}}(s_2)\backslash {\textsc {ls}}(s_3))$, or $c\in ({\textsc {ls}}(s_2)\backslash {\textsc {ls}}(s_4))$. In the first case, both must receive the message. In the latter cases, the listener receives and the non-listener discards. Accordingly, when a message is sent by one system, it is propagated to all other connected systems in a joint transition. A multicast is indeed blocking because a connected system cannot discard an incoming message on a channel it is listening to. More precisely, a joint transition $((s_1,s_2),(\upsilon ,!,c),(s'_1,s'_2))$ where $c\in {\textsc {ls}}(s_2)$ requires that $(s_2,(\upsilon ,?,c),s'_2)$ is supplied. In other words, message sending is blocked until all connected receivers are ready to participate in the interaction.

Consider now a broadcast. A broadcast is non-blocking. Thus, a broadcast message is either received or discarded. Suppose that a message originates from $\mathscr {T}_1$, i.e., $(s_1,(\upsilon ,!,\star ),s'_1)\in R_1$. If $\mathscr {T}_2$ is receiving, i.e., $(s_2,(\upsilon ,?,\star ),s'_2)\in R_2$ the message is sent. However, by definition, we have that $\star \in {\textsc {ls}}(s)$ for every *s* in a CTS. Namely, a system may not disconnect the broadcast channel $\star$. For this reason, the last part of the transition relation *R* defines a special case for handling (non-blocking) broadcast. Accordingly, a joint transition $((s_1,s_2),(\upsilon ,\gamma ,\star ),(s'_1,s'_2))\in R$ where $\gamma \in \{{!,?}\}$ is always possible and may not be blocked by any receiver. In fact, if ($\gamma =\ !$) and $(s_1,(\upsilon ,!,\star ),s'_1)\in R_1$ then the joint transition is possible whether $(s_2,(\upsilon ,?,\star ),s'_2)\in R_2$ or not. In other words, a broadcast can happen even if there are no receivers. Furthermore, if ($\gamma =\ ?$) and $(s_1,(\upsilon ,?,\star ),s'_1)\in R_1$ then also the joint transition is possible regardless of the other participants. In other words, a broadcast is received only by interested participants.

### Properties of parallel composition

Our parallel composition is commutative and associative. Furthermore, it supports non-blocking broadcast and blocking multicast semantics as stated in the following lemmas:

#### Lemma 1

(Commutativity and Associativity) Given two CTS $\mathscr {T}_1$ and $\mathscr {T}_2$ we have that:

- $$\Vert $$ is commutative: $$\mathscr {T}_1\Vert \mathscr {T}_2=\mathscr {T}_2\Vert \mathscr {T}_1$$;
- $$\Vert $$ is associative: $$(\mathscr {T}_1\Vert \mathscr {T}_2) \Vert \mathscr {T}_3=\mathscr {T}_1\Vert (\mathscr {T}_2 \Vert \mathscr {T}_3)$$.

Note that Lemma 1 is crucial to ensure that our parallel compostion operator is a commutative monoid, as otherwise it would not represent the right behaviour of interacting programs.

#### Lemma 2

(Non-blocking Broadcast) Given a CTS $\mathscr {T}_1$ and for every other CTS $\mathscr {T}$, we have that for every reachable state $(s_1,s)$ of $\mathscr {T}_1\Vert \mathscr {T}$ the following holds.

$$\begin{aligned} (s_1,(\upsilon ,!,\star ),s'_1)\in R_1\ \hbox {implies}\ ((s_1,s),(\upsilon ,!,\star ),(s'_1,s'))\in R_{\mathscr {T}_1\Vert \mathscr {T}} \end{aligned}$$

#### Lemma 3

(Blocking Multicast) Given a CTS $\mathscr {T}_1$ and a multicast channel $c\in C\backslash \{{\star }\}$ such that $(s_1,(\upsilon ,!,c),s'_1)\in R_1$, then for every other CTS $\mathscr {T}$ we have that in every reachable state $(s_1,s)$ of $\mathscr {T}_1\Vert \mathscr {T}$ the following holds.

The proofs of these lemmas are omitted here and included in the Appendix.

## ${\textsc {ReCiPe}}$: reconfigurable communicating programs

We formally present the ${\textsc {ReCiPe}}$ communication formalism and its main ingredients. We start by specifying agents (or programs) and their local behaviours. We give semantics to individual agents in terms of channelled transition systems (CTS). Therefore, we use the parallel composition operator in Definition 3 to compose the individual behaviour of the different agents to generate a global (or a system) one.

While the CTS semantics makes it clear what are the capabilities of individual agents and their interaction, it may not be the most convenient in order to mechanically analyse large systems comprised of multiple agents. Thus, we provide a symbolic semantics at system level using discrete systems. This second semantics enables efficient analysis by representing *closed* systems through the usage of BDDs or representing computations through Boolean formulas. We show that the two semantics (when restricted to *closed* systems) coincide.

The efficient analysis of *open* ${\textsc {ReCiPe}}$ systems is left as future work.

We assume that a set of *K* agents agree on a set of common variables ${\textsc {cv}}$, a set of data variables ${\textsc {d}}$, and a set of channels ${\textsc {ch}}$ containing the broadcast channel $\star$. As explained, common variables are variables that are owned (separately) by all agents. The values of these variables may be different in different agents. The common variables are used in order to have a common language to express properties that are interpretable on all agents (as either true or false).

### Definition 4

*(Agent)* An agent is $A_{i}=\langle V_{i},\ f_{i},\ {g}^{s}_{i},\ {g}^{r}_{i},$ , where:

- $V_{i}$ is a finite set of typed local variables, each ranging over a finite domain. A state ${s}^{i}_{}$ is an interpretation of $V_{i}$, i.e., if $\mathsf {Dom}(v)$ is the domain of *v*, then ${s}^{i}_{}$ is an element in $\prod _{v\in V_{i}}\mathsf {Dom}({v})$. We use $V'$ to denote the primed copy of *V* and $\mathsf {Id}_{i}$ to denote the assertion $\bigwedge _{v\in V_{i}}v=v'$.
- ${f_{i}}:{{\textsc {cv}}}\rightarrow {V_{i}}$ is a renaming function, associating common variables to local variables. We freely use the notation $f_{i}$ for the assertion $\bigwedge _{cv\in {\textsc {cv}}}cv=f_{i}(cv)$.
- ${g}^{s}_{i}(V_{i}, {\textsc {ch}}, {\textsc {d}}, {\textsc {cv}})$ is a send guard specifying a condition on receivers. That is, the predicate, obtained from ${g}^{s}_{i}$ after assigning ${s}^{i}_{}$, $ch$, and $\mathbf{d}$ (an assignment to ${\textsc {d}}$) , which is checked against every receiver *j* after applying $f_{j}$.
- ${g}^{r}_{i}(V_{i}, {\textsc {ch}})$ is a receive guard describing the connection of an agent to channel $ch$. We let ${g}^{r}_{i}(V_{i}, \star )$ $= \mathsf{true}$, i.e., every agent is always connected to the broadcast channel. We note, however, that receiving a broadcast message could have no effect on an agent.
is an assertion describing the send transition relation.
is an assertion describing the receive transition relation. We assume that agents are broadcast input-enabled, i.e., $$\forall v, \mathbf{d}\ \exists v'\ {{\mathbf {\mathsf{{s.t.}}}}}\ $$ .
  In examples, we use $\textsc {keep}(X)$ to denote that the variables *X* are not changed by a transition (either send or receive). More precisely, $\textsc {keep}(X)$ is equivalent to the following assertion $\bigwedge _{x\in X}x=x'$.
- $\theta _{i}$ is an assertion on $V_{i}$ describing the initial states, i.e., a state is initial if it satisfies $\theta _{i}$.

Agents exchange messages. A message (that we shall call *an observation*) is defined by the channel it is sent on (ch), the data it carries (**d**), the sender identity (i), and the assertion describing the possible local assignments to common variables of receivers ($\pi$). Formally:

### Definition 5

*(Observation)* An observation is a tuple $m=\left( ch,\mathbf{d},i,\pi \right)$, where $ch$ is a channel, $\mathbf{d}$ is an assignment to ${\textsc {d}}$, $i$ is an identity, and $\pi$ is a predicate over ${\textsc {cv}}$.

In Definition 5 we interpret $\pi$ as a set of possible assignments to common variables ${\textsc {cv}}$. In practice, $\pi$ is obtained from ${g}^{s}_{i}({s}^{i}_{},ch,\mathbf{d},{\textsc {cv}})$ for an agent $i$, where ${s}^{i}_{}\in \prod _{v\in V_{i}}\mathsf {Dom}({v})$ and $ch$ and $\mathbf{d}$ are the channel and assignment in the observation. We freely use $\pi$ to denote either a predicate over ${\textsc {cv}}$ or its interpretation, i.e., the set of variable assignments *c* such that $c \models \pi$. We also use $\pi (f^{-1}_{i}(s_{i}))$ to denote the assignment of $v\in {\textsc {cv}}$ by $s_{i}(f_{i}(v))$ in $\pi$.

The semantics of an agent $A_i$ is the CTS $\mathscr {T}(A_i)$ defined as follows.

### Definition 6

*(Agent Semantics)* Given an agent $A_i$ we define $\mathscr {T}(A_i)= \langle C,\varSigma ,\varUpsilon ,S,S_0,R,L,{\textsc {ls}}\rangle$, where the components of $\mathscr {T}(A_i)$ are as follows.

- $$C = {\textsc {ch}}$$
- $\varSigma = \prod _{v\in V_i} \mathsf {Dom}(v)$, i.e., the set of states of $A_i$
- $\varUpsilon = \varUpsilon ^+\times \{!,?\} \times {\textsc {ch}}$ and $\varUpsilon ^+ = 2^{{\textsc {d}}} \times K \times 2^{2^{{\textsc {cv}}}}$, where the set *K* ranges over the identities of the senders.
- $$S=\varSigma $$
- $$S_0 = \{ s\in S ~|~ \theta _i(s) \}$$
- $$R =$$
- $$L(s)=s$$
- $${\textsc {ls}}(s) = \{ c\in C ~|~ {g}^{r}_{i}(s,c)\}$$

Generally, the semantics of an agent is defined as an *open* CTS $\mathscr {T}(A_i)$. The transition alphabet $\varUpsilon$ of $\mathscr {T}(A_i)$ is the set of observations (as in Definition 5) that are additionally labelled with either send (!) or receive (?) symbols, corresponding to send and receive transitions. Furthermore, in every state *s*, an agent is listening to the set of channels in ${\textsc {ls}}(s)$. Namely, all channels that satisfy the agent’s receive guard ${g}^{r}_{i}$ in state *s*. We give further intuition for the definition of the transition relation *R*.

A triplet $(s,\upsilon ,s') \in R$, where $\upsilon =(\mathbf{d},i,\pi ,\gamma ,ch)$, if the following holds:

- Case ($\gamma =!$): Agent $i$ is a sender and we have that $\pi ={g}^{s}_{i}(s_{i},ch, \mathbf{d})$, i.e., $\pi$ is obtained from ${g}^{s}_{i}$ by assigning the state of $i$, the data variables assignment $\mathbf{d}$ and the channel $ch$, and  evaluates to $\mathsf{true}$.
- Case ($\gamma =?$): Agent $i$ is a receiver (potentially) accepting a message from another agent $i'$ on channel *c* and data $\mathbf{d}$ with a send guard $\pi$ such that $c\in {\textsc {ls}}(s)$, $\pi (f^{-1}_{i}(s_{i}))$, and . Note that the condition $i'\ne i$ is required to ensure that the message is sent by another agent.

Intuitively, if the agent $i$ is the sender, it determines the predicate $\pi$ (by assigning $s_{i}$, $\mathbf{d}$, and $ch$ in ${g}^{s}_{i}$) and $i$’s send transition  is satisfied by assigning $s_{i}$, $s'_{i}$, $\mathbf{d}$, and $ch$ to it. That is, upon sending the message with $\mathbf{d}$ on channel $ch$ the sender changes the state from $s_i$ to $s'_{i}$. If the agent $i$ is the receiver, it must satisfy the condition on receivers $\pi$ (when translated to its local copies of the common variables), it must be connected to $ch$ (according to ${g}^{r}_{i}$), and it must have a valid receive transition  when reading the data sent in $\mathbf{d}$ on channel $ch$.

Note that the semantics of an individual agent is totally decoupled from the semantics of how agents interact. Thus, different interaction modes (or parallel composition operators) can be adopted without affecting the semantics of individual agents. In our case, we have chosen to implement broadcast as a non-blocking send and non-blocking receive and a multicast as a blocking send and receive. However, if one chooses to do so, other composition operators could be defined. For example, a point-to-point composition would allow only two agents to communicate over a channel and would not allow send without receive.

A set of agents agreeing on the common variables ${\textsc {cv}}$, data variables ${\textsc {d}}$, and channels ${\textsc {ch}}$ define a *system*. We define a CTS capturing the interaction and then give a DS-like symbolic representation of the same system.

Let $S_{i}=\varPi _{v\in V_{i}}\mathsf {Dom}(v)$ be the set of states of agent $i$ and $S=\varPi _{i}S_{i}$ be the set of states of the whole system. Given an assignment $s\in S$ we denote by $s_{i}$ the projection of *s* on $S_{i}$.

### Definition 7

*(* ${\textsc {ReCiPe}}$ *System as a CTS)* Given a set $\{A_{i}\}_{i}$ of agents, a system is defined as the parallel composition of the CTS representations of all $A_{i}$, i.e., a system is a CTS of the form $\mathscr {T}=\Vert _{i\in I}\mathscr {T}(A_{i})$.

A triplet $(s,\upsilon ,s')$, where $\upsilon =(\mathbf{d},i,\pi ,!,c)$ is in the transition relation of the composed system $\mathscr {T}$ (according to Definition 3), if the following conditions hold:

- There exists a sender $i$ such that $(s_i,(\mathbf{d},i,\pi ,!,c),s'_i)\in R_{i}$. By Definition 6, we know that $(s_i,(\mathbf{d},i,\pi ,!,c),s'_i)\in R_{i}$ iff $\pi ={g}^{s}_{i}(s_{i},ch, \mathbf{d})$, i.e., $\pi$ is obtained from ${g}^{s}_{i}$ by assigning the state of $i$, the data variables assignment $\mathbf{d}$ and the channel $ch$, and  evaluates to $\mathsf{true}$.
- For every other agent $i'$ we have that either:
  1. $c\in {\textsc {ls}}^{i'}(s_{i'})$ and $(s_{i'},(\mathbf{d},i,\pi ,?,c),s'_{i'})\in R_{i'}$. By Definition 6, we know that $c\in {\textsc {ls}}^{i'}(s_{i'})$ and $(s_{i'},(\mathbf{d},i,\pi ,?,c),s'_{i'})\in R_{i'}$ iff ${g}^{r}_{i'}(s_{i'},c)$, $\pi (f^{-1}_{i'}(s_{i'}))$, and , all evaluate to $\mathsf{true}$;
  2. $c\notin {\textsc {ls}}^{i'}(s_{i'})$ and $s_{i'}=s'_{i'}$. By Definition 6 this is equivalent to $\lnot {g}^{r}_{i'}(s_{i'},ch)$; or
  3. $ch=\star$ and $s_{i'}=s'_{i'}$. By Definition 6 this is equivalent to $\lnot \pi (f^{-1}_{i'}(s_{i'}))$.

Intuitively, a message $(\mathbf{d},i,\pi ,!,c)$ labels a transition from *s* to $s'$ if the sender $i$ determines the predicate (by assigning $s_{i}$, $\mathbf{d}$, and $ch$ in ${g}^{s}_{i}$) and the send transition of $i$ is satisfied by assigning $s_{i}$, $s'_{i}$, $\mathbf{d}$, and $ch$ to it, i.e., the sender changes the state from $s_i$ to $s'_{i}$ and sets the data variables in the observation to $\mathbf{d}$. All the other agents either (a) satisfy this condition on receivers (when translated to their local copies of the common variables), are connected to $ch$ (according to ${g}^{r}_{i'}$), and perform a valid transition when reading the data sent in $\mathbf{d}$ on $ch$, (b) are not connected to $ch$ (according to ${g}^{r}_{i'}$) and all their variables do not change, or (c) the channel is a broadcast channel, the agent does not satisfy the condition on receivers, and all their variables do not change.

In order to facilitate symbolic analysis, we now define a symbolic version of $\Vert _{k\in K}\mathscr {T}(A_{k})$, under closed world assumption. That is, we only focus on messages that originate from the system under consideration. In fact, from an external observer point of view, only message sending is observable while reception cannot be observed. This notion of observability is the norm in existing theories on group communication [26, 51]. Thus, we consider the paths of $\Vert _{k\in K}\mathscr {T}(A_{k})$ that are of the form $\sigma =s_0,a_0,s_1,a_1,\ldots$ such that $a_j$ is of the form $(\mathbf{d},i,\pi ,!,c)$, $s_0\in S_0$ and for every $j\ge 0$ we have $(s_j,a_j,s_{j+1})\in R$. Note that $(\mathbf{d},i,\pi ,!,c)$ coincides with our definition of an observation *m*.

Thus, let $\varUpsilon$ be the set of possible observations in $\Vert _{k\in K}\mathscr {T}(A_{k})$. That is, let ${\textsc {ch}}$ be the set of channels, $\mathscr {D}$ the product of the domains of variables in ${\textsc {d}}$, *K* the set of agent identities, and $\varPi ({\textsc {cv}})$ the set of predicates over ${\textsc {cv}}$ then $\varUpsilon \subseteq {\textsc {ch}}\times \mathscr {D}\times K\times \varPi ({\textsc {cv}})$. In practice, we restrict attention to predicates in $\varPi ({\textsc {cv}})$ that are obtained from ${g}^{s}_{i}(V_{i},{\textsc {ch}},{\textsc {d}}, {\textsc {cv}})$ by assigning $V_{i}$ (a state of the agent with identity $i$), ${\textsc {ch}}$, and ${\textsc {d}}$.

Furthermore, we extend the format of the allowed transitions in the classical definition of a discrete system from assertions over an extended set of variables to assertions that allow quantification.

### Definition 8

*(Discrete System)* Given a set $\{A_{i}\}_{i}$ of agents, a system is defined as follows: $S=\langle {\mathscr {V},\ \rho ,\ \theta }\rangle$, where $\mathscr {V}=\biguplus \limits _{i}{V_i}$, a state of the system is in $\prod _{i}\prod _{v\in V_{i}}\mathsf {Dom}({v})$ and the initial assertion $\theta =\bigwedge \limits _{i}^{}{\theta _{i}}$. The transition relation of the system is characterised as follows:

The transition relation $\rho$ relates a system state ${s}_{}$ to its successors $s'$ given an observation $m=\left( ch,\mathbf{d},k,\pi \right)$. Namely, there exists an agent *k* that sends a message with data $\mathbf{d}$ (an assignment to ${\textsc {d}}$) with assertion $\pi$ (an assignment to ${g}^{s}_{k}$) on channel $ch$ and all other agents are either (a) connected, satisfy the send predicate, and participate in the interaction, (b) not connected and idle, or (c) do not satisfy the send predicate of a broadcast and idle. That is, the agents satisfying $\pi$ (translated to their local state by the conjunct $\exists {\textsc {cv}}.f_j$) and connected to channel $ch$ (i.e., ${g}^{r}_{j}({s}^{j}_{}, ch)$) get the message and perform a receive transition. As a result of interaction, the state variables of the sender and these receivers might be updated. The agents that are *not connected* to the channel (i.e., $\lnot {g}^{r}_{j}({s}^{j}_{}, ch)$) do not participate in the interaction and stay still. In case of broadcast, namely when sending on $\star$, agents are always connected and the set of receivers not satisfying $\pi$ (translated again as above) stay still. Thus, a blocking multicast arises when a sender is blocked until all *connected* agents satisfy $\pi \wedge f_j$. The relation ensures that, when sending on a channel that is different from the broadcast channel $\star$, the set of receivers is the full set of *connected* agents. On the broadcast channel agents who do not satisfy the send predicate do not block the sender.

The translation above to a transition system leads to a natural definition of a trace, where the information about channels, data, senders, and predicates is lost. We extend this definition to include this information as follows:

### Definition 9

*(System trace)* A system trace is an infinite sequence $\rho ={s}_{0}m_0,{s}_{1}m_1,\ldots$ of system states and observations such that $\forall t\ge 0$: $m_t=\left( ch_t,\mathbf{d}_t,k,\pi _t\right)$, $\pi _t={g}^{s}_{k}({s}^{k}_{t},\mathbf{d}_t, ch_t)$, and:

That is, we use the information in the observation to localize the sender *k* and to specify the channel, data values, and the send predicate.

The following theorem states a full abstraction property [47], namely that the CTS semantics of systems and their discrete counterpart define the same transition relation, under closed world assumption. That is, by considering the messages originating from the system under consideration as the only observations.

### Theorem 3

(Full abstraction) Given a set of ${\textsc {ReCiPe}}$ agents $\{A_{i}\}_{i}$, their discrete system representation, defined as $S=\langle {\mathscr {V},\ \rho ,\ \theta }\rangle$, is semantically equivalent to the parallel composition of their CTS representation, defined as $\mathscr {T}=\Vert _{i}\mathscr {T}(A_{i})$, under closed world assumption. More precisely,

- for every assignment *s* to system variables $\mathscr {V}$, it follows that: $\theta (s)$ iff $s\in S_0$;
- for all assignments *s* and $s'$ to variables in $\mathscr {V}$ and respectively in $\mathscr {V}'$ it follows that: $\rho (s,s')$ iff there exist assignment to data variables $\mathbf{d}$, a communication channel $ch$, and an agent *i* such that $(s,(\mathbf{d},i,\pi ,!,ch),s')\in R_{\mathscr {T}}$.

### Proof

We prove each statement separately.

- For *k* agents in the symbolic representation, $\theta$ characterises the set of system states $S'\subseteq \varPi _{i}S_{i}$ that satisfy the initial conditions of all agents, i.e., $\{s~|~s=(s_{0},s_{1},\dots ,s_{k}) \hbox {and} s\models \bigwedge \limits _{i}^{}{\theta _{i}}\}$. Note that $(s_{0},s_{1},\dots ,s_{k})\models \bigwedge \limits _{i}^{}{\theta _{i}}$ iff $s_{0}\models \theta _0\wedge s_{1}\models \theta _1\wedge \dots \wedge s_{k}\models \theta _k$. By Definitions 3 and 6 this is exactly the set of initial states $S_0$ in $\mathscr {T}=\Vert _{i}\mathscr {T}(A_{i})$;
- By Definition 8, we have that $\rho (s, s')$ evaluates to true if there exists a valuation $\mathbf{d}$ to ${\textsc {d}}$ and a channel $ch$ in ${\textsc {ch}}$ such that both of the following hold:
  - There exists an agent $i$ such that the send transition  is satisfied by assigning to current *local* state $s_i$, next local state $s'_i$ (i.e., the projection of the system states *s* and $s'$ on agent $i$), the valuation $\mathbf{d}$, and the communication channel $ch$. According to the enumerative semantics in Definition 6, agent $i$ has an individual send transition given the current *local* state $s_{i}$, next local state $s'_{i}$, valuation $\mathbf{d}$ to data variables, and channel $ch$. Namely, agent $i$ has a send transition $(s_i,(\mathbf{d},i,\pi ,!,ch),s'_i)\in R_{i}$ such that $\pi ={g}^{s}_{i}(s_{i},ch, \mathbf{d})$, i.e., $\pi$ is obtained from ${g}^{s}_{i}$ by assigning the state of $i$, the data variables assignment $\mathbf{d}$ and the channel $ch$, and  evaluates to $\mathsf{true}$.
  - For every other agent $i'$ we have that either:
    1. it is connected to channel $ch$ (i.e., ${g}^{r}_{i'}(s_{i'},ch)$ holds), satisfies the send predicate (i.e., $\pi (f^{-1}_{i'}(s_{i'}))$ holds), and participates in the interaction (i.e.,  holds). By Definition 6, we know that agent $i'$ has an individual receive transition $(s_{i'},(\mathbf{d},i,\pi ,?,ch),s'_{i'})\in R_{i'}$ where $ch\in {\textsc {ls}}^{i'}(s_{i'})$;
    2. it is not connected to channel $ch$ (i.e., $\lnot {g}^{r}_{i'}(s_{i'},ch)$ ) and $s_{i'}=s'_{i'}$. By Definition 6, agent $i'$ does not have a receive transition for this message. In other words, since $ch\notin {\textsc {ls}}^{i'}(s_{i'})$ then agent $i'$ cannot observe this transmission ;
    3. or the message is sent on a broadcast channel ($ch=\star$), where agent $i'$ does not satisfy the sender predicate (i.e., $\lnot \pi (f^{-1}_{i'}(s_{i'}))$) and $s_{i'}=s'_{i'}$. By Definition 6 this is equivalent to ignoring this message by not implementing a receive transition.
    So far, we have shown that every individual (send/receive transition) in the symbolic model has a corresponding one in the enumerative semantics of individual agents. We need to show that the composition of these individual transitions according to $\rho$ in the symbolic model has exactly the same semantics of the parallel composition in Definition 3. That is, $\rho (s, s')$ iff for the identified $\mathbf{d}$, *i*, $ch$ and $\pi$ we have $(s,(\mathbf{d},i,\pi ,!,ch),s')\in R_{\mathscr {T}}$, given the assignments *s* and $s'$ to variables in $\mathscr {V}$ and respectively in $\mathscr {V}'$.
    The existential quantification on sender transitions in $\rho$ (i.e., ) implies that the order of the composition is immaterial, namely any two systems states $(s_0,s_1,\dots ,s_k)$ and $(s_1,s_0,\dots ,s_k)$ that only differ in the order of individual agent’ states are semantically equivalent. By Lemma 1, we have that parallel composition is commutative, and thus the order is immaterial under the enumerative system semantics as well. If $\rho (s,s')$ is due to a message exchange on the broadcast channel $\star$ then the non-blocking semantics of the broadcast is preserved by the transition relation of the CTS composition as stated in Lemma 2. Moreover, if $\rho (s, s')$ is due to a message exchange on a multicast channel *c* then the blocking semantics of the multicast is preserved by the transition relation of the CTS composition as stated in Lemma 3. Lastly, the universal quantification on all possible receivers in $\rho$ (i.e., $\bigwedge \limits _{j\ne k}^{}{}$) follows by the CTS semantics of parallel composition in Definition 3, where a receive transition can be received jointly by different agents, and by the commutativity and associativity of parallel composition (Lemma 1), where the scope of a send transition can be extended to cover all possible receivers.
    The other direction of the proof follows in similar manners.

$$\square $$

The following is a corollary of Theorem 3 to relate the traces arising from Definition 9 to that of Definition 7.

### Corollary 1

(Trace equivalence) The traces of a symbolic system composed of a set of agents $\{A_{i}\}_{i}$ are the paths of the induced CTS.

## Reconfigurable manufacturing scenario

We complete the details of the RMS example, informally described in Sect. 2. Many aspects of the example are kept simple on purpose to aid the presentation.

The system, in our scenario, consists of an assembly product line agent () and several types of task-driven robots. We describe the behaviour of the product line and only robots of type-1 () as these are sufficient for exposing all features of ${\textsc {ReCiPe}}$.

A product line is responsible for assembling the main parts and delivering the final product. Different types of robots are responsible for sub-tasks, e.g., retrieving and/or assembling individual parts. The product line is generic and can be used to produce different products and thus it has to determine the set of resources, to recruit a team of robots, to split tasks, and to coordinate the final stage of production.

Every agent has copies of the common variables: ${\scriptstyle @\mathsf {type}}$ indicating its type (e.g., , , , ), ${\scriptstyle @\mathsf {asgn}}$ indicating whether a robot is assigned, and ${\scriptstyle @\mathsf {rdy}}$ indicating what stage of production the robot is in. The set of channels includes the broadcast channel $\star$ and multicast channels $\{\mathsf {A},\ldots \}$. For simplicity, we only use the multicast channel $\mathsf {A}$ and fix it to the line agent. The set of data variables includes ${\textsc {msg}}, {\textsc {no}},$ and ${\textsc {lnk}}$, indicating the type of the message, a number (of robots per type), and a name of a channel respectively.

We note that when a data variable is not important for some message it is omitted from the description of the message.

**Fig. 1:**
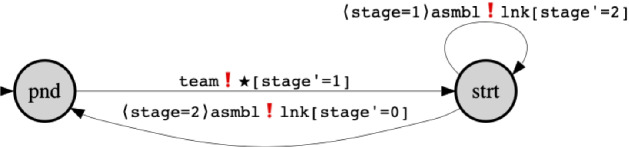
Product line agent

We start with the description of the line agent . We give a high-level overview of the protocol applied by the line agent using the state machine in Fig. 1. The states capture a partial evaluation of the state variables of the agent. In this case, the value of the state variable $\mathsf {st}$. Transitions labels represent guarded commands. We use the format “” to denote a guarded command $\mathsf {cmd}$. Namely, the predicate ${\varvec{\varPhi }}$ is a condition on the current assignment to local variables of an agent (and for receive transitions also on data variables that appear in the message). We freely use $\mathbf{d}$ to refer to an assignment to data variables. Usually, we write directly only the value of the ${\textsc {msg}}$ variable to avoid cluttering. Sometimes, we add the values of additional data variables. Each guarded command is labelled with a role ( for send and  for receive transitions); also with a channel name $\mathsf {ch}$ and a new assignment to local variables $\mathsf {[}\mathsf {v}'_\mathsf {1}=\mathsf {a}_\mathsf {1};\dots \mathsf {v}'_\mathsf {n}= \mathsf {a}_\mathsf {n]}$ to represent the side effects of the interaction. For the line agent, the protocol consists of starting from the pending state and sending a team formation broadcast. This is followed by sending of an assembly multicast on the channel stored in local variable $\mathsf {lnk}$ and updating the stage to 2. Finally, an additional assembly multicast on the same channel resets the process. We include below the full description with the guards and predicates. Each transition in the state machine corresponds to a disjunct in either the send or the receive transition predicate below. Variables that are not assigned in a transition are kept unchanged in the predicate. The send and receive guards of the agent are only partially captured in the state machine.

We now turn to the formal description of the line agent, starting with its set of variables. In addition to copies of common variables (e.g., $f_l({\scriptstyle @\mathsf {type}})$ $=\mathsf {ltype}$), the line agent has the following state variables: $\mathsf {st}$ is a state ranging over  (pending and start), $\mathsf {lnk}$ is the link of the product line, $\mathsf {prd}$ is the id of the active product, and $\mathsf {stage}$ is used to range over the different stages of production.

The initial condition ${\theta _l}$ of a line agent is defined as follows:Thus, starting from the pending state, the line agent has a task of assembling one of two products, and uses a multicast channel $\mathsf {A}$ to coordinate the assembly team. If there are multiple product lines, then each is initialised with a dedicated channel.

The send guard of the  agent is of the following form:Namely, broadcasts are sent to robots whose ${\scriptstyle @\mathsf {asgn}}$ is false (i.e., free to join a team). If the identity of the product to be assembled is 1, then the required agents are of types  and  and if the identity of the product is 2, then the required agents are of types  and . Messages on channel $\mathsf {A}$ (the value of $\mathsf {lnk}$) are sent to connected agents when they reach a matching stage of production, i.e., ${\scriptstyle @\mathsf {rdy}}=\mathsf {stage}$. The receive guard of  is $ch=\star$, i.e., it is only connected to channel $\star$.

We may now proceed by explaining ${\textsc {ReCiPe}}$ ’s send and receive transition relations of the line agent in light of the state machine in Fig. 1. The send transition relation of  is of the following form:The  agent starts in the pending state (see $\theta _l$). It broadcasts a request () for two robots () per required type asking them to join the team on the multicast channel stored in its $\mathsf {lnk}$ variable ($\mathbf{d}({\textsc {lnk}}\mapsto \mathsf {lnk})$). According to the send guard, described before, if the identity of the product to assemble is 1 () the broadcast goes to type 1 and type 2 robots and if the identity is 2 then it goes to type 1 and type 3 robots. Thanks to channel mobility (i.e., $\mathsf {\mathbf{d}({\textsc {lnk}})=lnk}$) a team on a dedicated link can be formed incrementally at run-time. As a side effects of broadcasting the  message, the line agent moves to the start state  where the first stage of production begins . In the start state, the line agent attempts an ${\textsc {assemble}}$ (blocking) multicast on $\mathsf {A}$. The multicast can be sent only when the entire team completed the work on the production stage (when their common variable ${\scriptstyle @\mathsf {rdy}}$ agrees with $\mathsf {stage}$ as specified in the send guard). One multicast increases the value of $\mathsf {stage}$ and keeps  in the start state. A second multicast finalises the production and  becomes free again.

We set  as ’s receive transition relation. That is,  is not influenced by incoming messages.

**Fig. 2:**
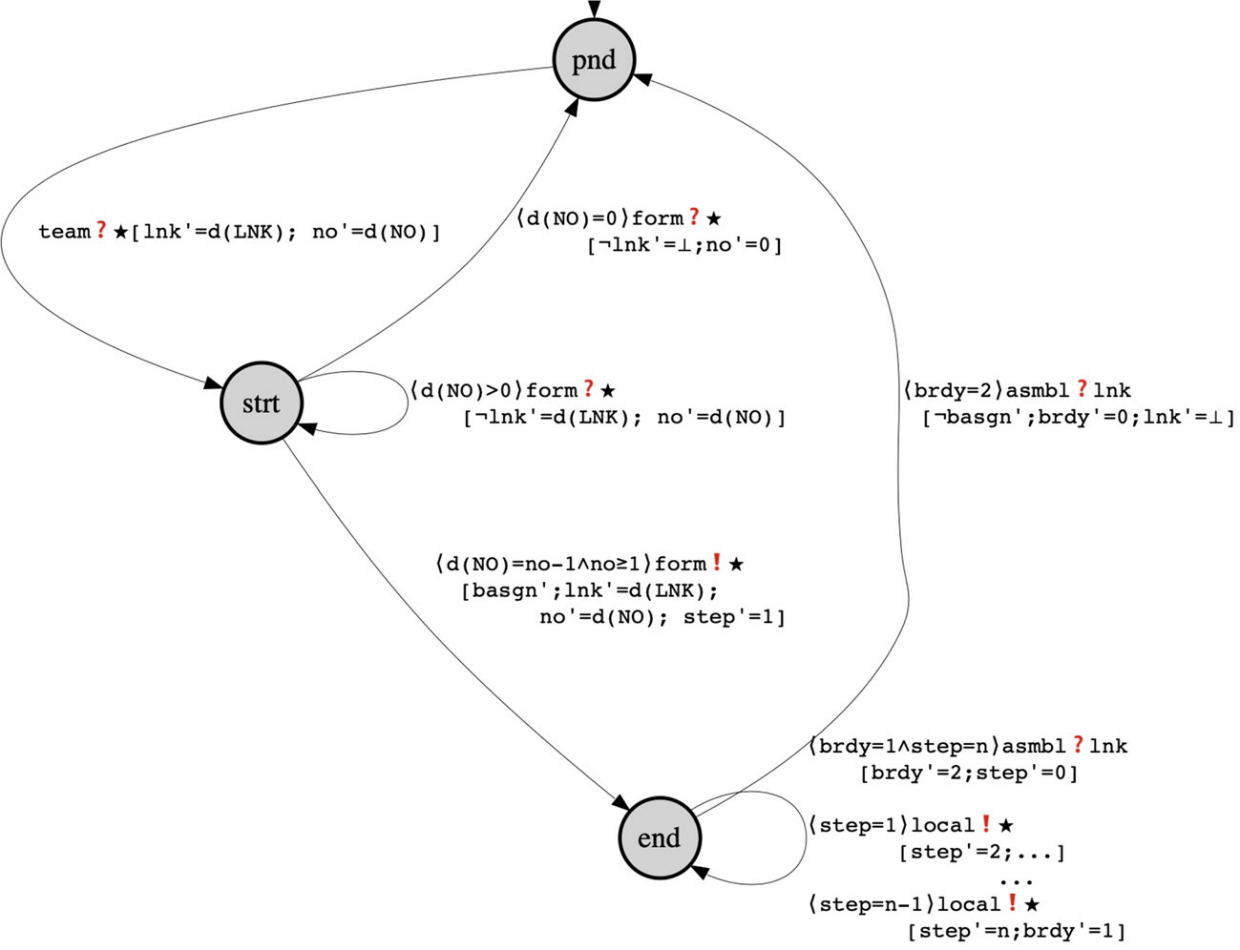
The agent of -robot

We now specify the behaviour of -robots and show how an autonomous and incremental one-by-one team formation is done anonymously at run-time. As before, we give a high-level overview of the protocol using the state machine in Fig. 2. The team formation starts when unassigned robots are in pending states (). From this state they may only receive a team message from a line agent. The message contains the number of required robots $\mathbf{d}({\textsc {no}})$ and a team link $\mathbf{d}({\textsc {lnk}})$. The robots copy these values to their local variables (i.e., $\mathsf {lnk}'=\mathbf{d}({\textsc {lnk}})$ etc.) and move to the start state (). From the start state there are three possible transitions:

- Join - move to state  - a robot joins the team by *broadcasting* a  message to -robots forwarding the number of still required robots () and the team link ($\mathsf {\mathbf{d}({\textsc {lnk}})=lnk}$). This message is sent only if , i.e, at least one robot is needed. From state  the robot starts its mission.
- Wait - stay in state  - a robot *receives* a  message from a robot, updating the number of still required robots (i.e., if ).
- Step back - return to state  - a robot *receives* a  message from a robot, informing that no more robots are needed, i.e., . The robot disconnects from the team link, i.e., 
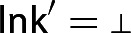
. Thus it may not block interaction on the team link.

After joining the team, a robot in state  (i.e., with ) starts its mission independently until it finishes (). We have used ($\dots$) to abstract the individual behaviour of the robot in state (). In fact, each local step corresponds to a broadcast message ($\mathsf {local}$) that is hidden from other agents. This will be clarified later in the send guard of the robot which evaluates to false when ($\mathsf {local}$) is enabled.

When all team robots finish their individual tasks (i.e., circled in the self-loop on state  while $\mathsf {brdy}=1$ until $\mathsf {step}=n$), they become ready to receive an  message on $\mathsf {A}$, to start the next stage of production (i.e, $\mathsf {brdy}'=2$) while still staying in  state.

From this final stage (i.e., ) the robots are ready to receive the final  message to finalise the product and subsequently they reset to their initial conditions.

As before, each transition corresponds to a disjunct in the send and receive transition relations, which are fully specified later in this section.

We now turn to the formal description of the robot, starting with its set of variables. In addition to copies of common variables a -robot has the following variables: $\mathsf {st}$ ranges over , $\mathsf {step}$ is used to control the progress of individual behaviour, $\mathsf {no}$ (resp. $\mathsf {lnk}$) is a placeholder to a number (resp. link) learned at run-time, and $f_b$ relabels common variables as follows: $f_b({\scriptstyle @\mathsf {type}})=\mathsf {btype}$, $f_b({\scriptstyle @\mathsf {asgn}})=\mathsf {basgn}$ and $f_b({\scriptstyle @\mathsf {rdy}})=\mathsf {brdy}$.

Initially, a robot is in the pending state and is available for recruitment:The send guard of the robot is of the following form:

$$\begin{aligned} \begin{array}{lr} {g}^{s}_{b} : (ch=\star )\wedge \mathbf{d}({\textsc {msg}}\ne \mathsf {local})\wedge ({\scriptstyle @\mathsf {type}}=\mathsf {btype})\wedge \lnot {\scriptstyle @\mathsf {asgn}}\ \vee \\ \qquad \qquad \qquad \qquad (ch=\star )\wedge \mathbf{d}({\textsc {msg}}=\mathsf {local})\wedge ({\scriptstyle @\mathsf {asgn}}\wedge \lnot {\scriptstyle @\mathsf {asgn}}) \end{array} \end{aligned}$$

Interestingly, the send guard delimits the scope of the broadcast, depending on the assignment to data variables. Namely, it specifies that a robot either broadcasts to unassigned robots of the same type if the message is not a local one ($\mathbf{d}({\textsc {msg}}\ne \mathsf {local}$) or otherwise hides the message from all other agents by broadcasting on a false predicate (i.e., the predicate ${\scriptstyle @\mathsf {asgn}}\wedge \lnot {\scriptstyle @\mathsf {asgn}}$). Note that such message cannot be received by any agent, and it can be regarded as a local computation. Thus, it becomes very easy to distinguish the individual behaviour of an agent from its interactions with the rest of the system.

The receive guard specifies that a -robot is connected either to a broadcast $\star$ or to a channel matching the value of its link variable:

$$\begin{aligned} \begin{array}{rcl} {g}^{r}_{b} : ch=\star \vee ch=\mathsf {lnk}. \end{array} \end{aligned}$$

Finally, we report the send  and receive  transition predicates below.

## LTOL: an extension of LTL

We introduce ltol, an extension of ltl with the ability to refer and therefore reason about agents interactions. We replace the next operator of ltl with the observation descriptors: *possible* $\langle {O}\rangle$ and *necessary* $[{O}]$, to refer to messages and the intended set of receivers. The syntax of formulas $\phi$ and *observation descriptors* *O* is as follows:We use the classic abbreviations $\mathbin {\rightarrow },\mathbin {\leftrightarrow }$ and the usual definitions for $\mathsf{true}$ and $\mathsf{false}$. We also introduce the temporal abbreviations  (*eventually*), $\mathsf {{G}}\phi \equiv \lnot \mathsf {{F}}\lnot \phi$ (globally) and  (*weak until*). Furthermore we assume that all variables are Boolean because every finite domain can be encoded by multiple Boolean variables. For convenience we will, however, use non-Boolean variables when relating to our RMS example.

The syntax of ltol is presented in *positive normal form* to facilitate translation into alternating Büchi automata (ABW) as shown later. That is, we push the negation down to atomic propositions. We, therefore, use $\overline{\varTheta }$ to denote the dual of formula $\varTheta$ where $\varTheta$ ranges over either $\phi$ or *O*. Intuitively, $\overline{\varTheta }$ is obtained from $\varTheta$ by switching $\vee$ and $\wedge$ and by applying dual to sub formulas, e.g., , $$\overline{\phi _1 \wedge \phi _2} = \overline{\phi _1} \vee \overline{\phi _2},\ $$ $\overline{cv} = \lnot cv$, and $\overline{\bullet ^{\exists }{O}} = \bullet ^{\forall }{\overline{O}}$.

Observation descriptors are built from referring to the different parts of the observations and their Boolean combinations. Thus, they refer to the channel in ${\textsc {ch}}$, the data variables in ${\textsc {d}}$, the sender *k*, and the predicate over common variables in ${\textsc {cv}}$. These predicates are interpreted as sets of possible assignments to common variables, and therefore we include existential $\bullet ^{\exists }{O}$ and universal $\bullet ^{\forall }{O}$ quantifiers over these assignments.

The semantics of an observation descriptor *O* is defined for an observation $m = \left( ch,\ \mathbf{d},\ k,\ \pi \right)$ as follows:

$$\begin{aligned} \begin{array}{lcc} m \models cv\quad \mathbf{iff} \quad \text {for every assignment}\ c \models \pi \ \text {we have}\ c \models cv&{}\\ m \models \lnot cv\ \ \mathbf{iff} \ \ \text {there is an assignment}\ c \models \pi \ \text {such that}\ c \not \models cv&{}\\ m \models \bullet ^{\exists }{O} \ \ \mathbf{iff} \ \ \text {there is an assignment}\ c \models \pi \ \text {such that}\ \left( ch, d, k, \{c\}\right) \models O&{}\\ m \models \bullet ^{\forall }{O} \ \ \mathbf{iff} \ \ \text {for every assignment}\ c \models \pi \ \text {it holds that}\ \left( ch, d, k, \{c\}\right) \models O&{}\\ m \models O_1 \vee O_2 \quad \mathbf{iff} \quad \text {either}\ m \models O_1\ \text {or}\ m \models O_2&{}\\ m \models O_1 \wedge O_2 \quad \mathbf{iff} \quad m \models O_1\ \text {and}\ m \models O_2&{} \end{array} \end{aligned}$$

We only comment on the semantics of the descriptors $\bullet ^{\exists }{O}$ and $\bullet ^{\forall }{O}$ as the rest are standard propositional formulas. The descriptor $\bullet ^{\exists }{O}$ requires that at least one assignment *c* to the common variables in the sender predicate $\pi$ satisfies *O*. Dually $\bullet ^{\forall }{O}$ requires that all assignments in $\pi$ satisfy *O*. Using the former, we express properties where we require that the sender predicate has a possibility to satisfy *O* while using the latter we express properties where the sender predicate can only satisfy *O*. For instance, both observations $\left( ch, \mathbf{d}, k, cv_1\vee \lnot cv_2\right)$ and $\left( ch, \mathbf{d}, k, cv_1\right)$ satisfy $\bullet ^{\exists }{cv_1}$ while only the latter satisfies $\bullet ^{\forall }{cv_1}$. Furthermore, the observation descriptor $\bullet ^{\forall }{\mathsf{false}}\wedge ch=\star$ says that a message is sent on the broadcast channel with a false predicate. That is, the message cannot be received by other agents. In our RMS example in Sect. 6, the descriptor  says that the message is intended exactly for robots of type-1.

Note that the semantics of $\bullet ^{\exists }{O}$ and $\bullet ^{\forall }{O}$ (when nested) ensures that the outermost cancels the inner ones, e.g., $\bullet ^{\exists }{(O_1\vee (\bullet ^{\forall }{(\bullet ^{\exists }{O_2})}))}$ is equivalent to $\bullet ^{\exists }{(O_1\vee O_2)}$. Furthermore, when $cv$ and respectively $\lnot cv$ appear outside the scope of a quantifier ($\bullet ^{\forall }{}$ or $\bullet ^{\exists }{}$), they are semantically equivalent to the descriptors $\bullet ^{\forall }{cv}$ and respectively $\bullet ^{\exists }{\lnot cv}$. Thus, we assume that they are written in the latter normal form.

We interpret ltol formulas over system computations:

### Definition 10

*(System computation)* A system computation $\rho$ is a function from natural numbers *N* to $2^{\mathscr {V}}\times M$ where $\mathscr {V}$ is the set of state variable propositions and $M={\textsc {ch}}\times 2^{{\textsc {d}}}\times K\times 2^{2^{{\textsc {cv}}}}$ is the set of possible observations. That is, $\rho$ includes values for the variables in $2^{\mathscr {V}}$ and an observation in *M* at each time instant.

We denote by ${s}_{i}$ the system state at the *i*th time point of the system computation. Moreover, we denote the suffix of $\rho _{}$ starting with the *i*th state by $\rho _{\ge i}$ and we use $m_{i}$ to denote the observation $\left( ch, \mathbf{d}, k, \pi \right)$ in $\rho _{}$ at time point *i*.

The semantics of an ltol formula $\varphi$ is defined for a computation $\rho _{}$ at a time point $i$ as follows:Intuitively, the temporal formula $\langle {O}\rangle \phi$ is satisfied on the computation $\rho _{}$ at point $i$ if the observation $m_i$ satisfies *O* *and* $\phi$ is satisfied on the suffix computation $\rho _{\ge i+1}$. On the other hand, the formula $[{O}]\phi$ is satisfied on the computation $\rho _{}$ at point $i$ if $m_i$ satisfying *O* *implies* that $\phi$ is satisfied on the suffix computation $\rho _{\ge i+1}$. Other formulas are interpreted exactly as in ltl.

With observation descriptors we can refer to the intention of agents in the interaction. For example, the following descriptorspecifies that the target of the message is “exactly and only" type-1 and type-2 robots. This descriptor can be used later to specify that whenever the line agent “*l*" recruits for a product with identity 1, it notifies both type-1 and type-2 robots as follows:Namely, whenever the line agent is in the pending state and tasked with product 1 it notifies both type-1 and type-2 robots by a broadcast.

The pattern “After *q* have exactly two *p* until *r*” [24, 45] can be easily expressed in ltl and can be used to check the formation protocol. Consider the following formulas:specifying that a team message is sent to type-1 robots and requires two robots,specifying that a formation message is sent to type-1 robots, and

$$\begin{aligned} \varphi _3:=\langle {ch=\mathsf {A}}\rangle \mathsf{true}\end{aligned}$$

specifying that a message is sent on channel $\mathsf {A}$.

Now, the template “After $\varphi _1$ have exactly two $\varphi _2$ until $\varphi _3$” specifies that whenever a team message is sent to robots of type-1 requiring two robots, then two form messages destined for type-1 robots will follow before using the multicast channel. That is, two type-1 robots join the team before a (blocking) multicast on channel $\mathsf {A}$ may become possible.

We can also reason at a local rather than a global level. For instance, we can specify that robots follow a “correct” utilisation of channel $\mathsf {A}$. Formally,specifies that a team message is sent to robots of type ;specifies that a robot different from *k* sends a form message specifying that no more robots are needed and this message is sent to unassigned type  robots;specifies that an assembly message is sent on channel $\mathsf {A}$ to robots of type  who reached stage 2 of the production. Thus, for robot *k* of type , the formulas1state that: (i) robots are not connected to channel $\mathsf {A}$ until they get a team message, inviting them to join a team; (ii) if either they are not selected ($O_2(k,t)$) or they finished production after selection ($O_3(t)$) then they disconnect again until the next team message. This reduces to checking the “correct” utilisation of channel $\mathsf {A}$ to individual level, by verifying these properties on all types of robots independently. By allowing the logic to relate to the set of targeted robots, verifying all targeted robots separately entails the correct “group usage" of channel *A*.

### The satisfiability and the model checking problems of ltol

In this section, we improve our early results on satisfiability and model checking of ltol, presented in the ${\textsc {aamas}}$ version [1] of this article. In that version, we computed an expspace upper bound for both problems with respect to the set of common variables ${\textsc {cv}}$ that appear in the observation descriptors and pspace upper bound with respect to the rest of the input. This result was not surprising as the semantics of observations requires quantification on the assignments to common variables ${\textsc {cv}}$ appearing in *O*. Indeed, the number of assignments to ${\textsc {cv}}$ is doubly exponential in the size of ${\textsc {cv}}$, i.e, the number of assignments is $2^{2^{|{{\textsc {cv}}}|}}$. Both problems require translation to Nondeterministic Büchi Automata (NBW), and a direct translation would incur a double exponential blowup in the size of the automaton with respect to $|{{\textsc {cv}}}|$. Thus, a membership in expspace with respect to $|{{\textsc {cv}}}|$ follows from the membership in nlogspace of the nonemptiness problem for NBW.

In this article, we improved the latter results to pspace, matching the lower bound. This is achieved by a novel automaton construction. Namely, we introduce a further dependency between the formula and the alphabet that is read by the automaton. Thus, the automaton does not read concrete messages but it rather partitions messages into sets, according to their effects on the truth values of subformulas of the formula.

Before we proceed with the automaton construction, we fix the sets of system variables $\mathscr {V}$, the communication channels ${\textsc {ch}}$, the data variables ${\textsc {d}}$, the identities of agents *K*, and the common variables ${\textsc {cv}}$.

Our direct construction in [1] considers a *state-alphabet* $\varSigma =2^{\mathscr {V}}$ and a *message-alphabet* $M={\textsc {ch}}\times {{\textsc {d}}} \times K \times 2^{2^{{\textsc {cv}}}}$. Clearly, the message-alphabet is doubly-exponential in ${\textsc {cv}}$ and implies that the decision procedures based on *M* would be in expspace (with respect to ${\textsc {cv}}$). However, *M* is “too large” for the automaton (c.f., [62]). Thus, we consider a smaller alphabet that is derived from the observation descriptors appearing in the formula. This alphabet is at most exponential in the size of the formula (allowing for pspace analysis). To achieve pspace analysis, we have to extend the decision procedures to further consider *observation-alphabet satisfiability* and *observation-alphabet model-checking*, as we will see below.

Recall the alphabets $\varSigma$ and *M* above and fix an ltol formula $\varphi$. Let ${\mathsf {obs}({\varphi })}$ be the set of observations appearing “top-level” in the operators $\langle {\cdot }\rangle$ and $[{\cdot }]$ in $\varphi$. More precisely, ${\mathsf {obs}({\varphi })}$ is closed under the subformula relation of $\varphi$, but is not closed under the subformula relation of *O*. Consider $\varphi _2(k,t)$ in Eq. 1:

$$\begin{aligned} {\mathsf {obs}({\varphi _2(k,t)})} =\{{O_2(k,t)\vee O_3(t), O_1(t)}\} \end{aligned}$$

We denote by $|{{\mathsf {obs}({\varphi })}}|$ the size of the set ${\mathsf {obs}({\varphi })}$. We denote by $|{O}|$ the length of the observation *O* and by $|{{\mathsf {obs}({\varphi })}}|$ the sum of lengths of observations in $\varphi$. Note that $|{{\mathsf {obs}({\varphi })}}|$ is bounded by the size of $\varphi$. Thus, we may now define an *observation-alphabet* , that is at most exponential in the size of $\varphi$. We will use this alphabet to enable pspace analysis.

In our construction, the automaton reads words from the alphabet  while system computations are derived from the alphabet $(\varSigma \times M)^\omega$.

Intuitively, an automaton word  and a system computation $\rho \in (\varSigma \times M)^\omega$ agree on the state-alphabet $\varSigma$ and only differ in their treatment to messages. Formally, given a word , and a system computation $\rho = (\sigma '_0,m_0),(\sigma '_1,m_1),\dots$. We say that $\rho$ satisfies *w* if for every $i\ge 0$ we have that $\sigma _i'=\sigma _i$ and for every $O\in {\mathsf {obs}({\varphi })}$ we have $m_i\models O$ iff . Note that $m_i\models O$ follows the semantics of observation descriptors. Thus, a word *w* defines a language over system computations.

More precisely, for a word  we denote by $L_{\omega }({w})$ the set of system computations satisfying *w*. We say that *w* is *non empty* if there is some system computation satisfying it, i.e., if $L_{\omega }({w})\ne \emptyset$. Furthermore, for a letter , we denote by  the set of models of . That is, all the messages that satisfy all the observations in  and do not satisfy all the observations that are not in . We say that  is *non empty* if .

Clearly, a word  is non empty if and only if for every $i\ge 0$ we have that  is non empty.

We show that satisfiability of ltol can be reduced to finding a word *w* such that the set of system computations satisfying *w* is not empty. Similarly, model checking is reduced to building an automaton for $\lnot \varphi$ and identifying a word *w* satisfying $\lnot \varphi$ and a computation $\rho$ of the system under study such that $\rho$ satisfies *w*.

The following theorem states that the set of computations satisfying a given formula are exactly the ones satisfying words accepted by some finite automaton on infinite words.

#### Theorem 4

For every ltol formula $\varphi$, there is an Alternating Büchi Automaton (ABW)  $q_0, F\subseteq Q \rangle$ such that $\bigcup _{w \in L_{\omega }({A_{\varphi }})} L_{\omega }({w})$ is exactly the set of computations satisfying the formula $\varphi$.

Notice that for a given word *w*, either *all* the computations that satisfy *w* satisfy $\varphi$ or *all* the computations that satisfy *w* do not satisfy $\varphi$ (i.e., satisfy $\overline{\varphi }$). In the first case *w* is accepted by $A_{\varphi }$ and in the second it is not accepted by $A_{\varphi }$. Thus, the definition of  is such that words partition the computations to equivalence sets that are uniform with respect to the satisfaction of $\varphi$.

#### Proof

The set of states *Q* is the set of all sub formulas of $\varphi$ with $\varphi$ being the initial state $q_0$. The automaton has two alphabets, namely the state-alphabet $\varSigma = 2^{\mathscr {V}}$ and the observation alphabet . The set *F* of accepting states consists of all sub formulas of the form . The transition relation  is defined inductively on the structure of $\varphi$, as follows:

if $v\in \sigma$ and $\mathsf{false}$ otherwise;
if $v \not \in \sigma$ and $\mathsf{false}$ otherwise;
;
;
;
;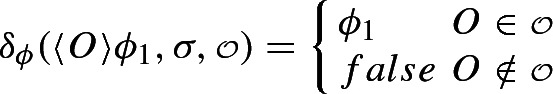;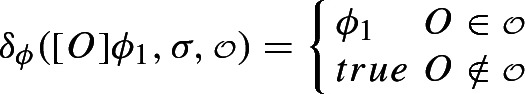.

The proof of correctness of this construction proceeds by induction on the structure of $\varphi$.

We prove that when $A_{\varphi }$ is in state $\phi _1$, it accepts exactly all computations that satisfy $\phi _1$. The base cases (i.e., state variable propositions) follow from the definition of $\delta _{\phi }$ while other cases follow from the semantics of $\varphi$ and the induction hypothesis. The construction ensures that a computation can only satisfy , if it has a suffix satisfying $\phi _2$; otherwise $A_{\phi }$ will have an infinite path stuck in  which is not accepting. $\square$

Note that, from Theorem 4, the number of states in $A_{\varphi }$ is linear in the size of $\varphi$, i.e., $|{Q}|$ is in $\mathscr {O}({|{\varphi }|})$. The size of the transition relation $|{\delta _{\phi }}|$ is in , i.e., it is in ${|{\varphi }|^2.2^{\mathscr {O}({|{\varphi }|})}}$. Finally, the size of the alternating automaton $|{A_{\varphi }}|$ is in $\mathscr {O}({|{Q}|.|{\delta _{\phi }}|})$, i.e., $|{A_{\varphi }}|$ is in $|{\varphi }|^3.2^{\mathscr {O}({|{\varphi }|})}$.

By Theorem 4 and Proposition 2, we have that:

#### Corollary 2

For every formula $\varphi$ there is an NBW $N_\varphi$ with a state-alphabet $\varSigma =2^{\mathscr {V}}$ and an observation-alphabet  where  and $\bigcup _{w \in L_{\omega }({N_{\varphi }})} L_{\omega }({w})$ is exactly the set of computations satisfying $\varphi$ such that:

- $|{Q}|$ is in $2^{\mathscr {O}({|{\varphi }|})}$ and $|{\delta }|$ is in , i.e., $|{\delta }|$ is in $2^{\mathscr {O}({|{\varphi }|})}$.
- The required space for building the automaton is $\textsc {nlog} {(|{Q}|.|{\delta }|)}$, i.e., it is in $\mathscr {O}({|{\varphi }|})$
- The size of the Büchi automaton is $|{Q}|.|{\delta }|$, i.e., $|{N}|$ is in $2^{\mathscr {O}({|{\varphi }|})}$.

#### Theorem 5

The satisfiability problem of ltol is pspace-${\textsc {complete}}$ with respect to $|{\varphi }|$.

#### Proof

By Corollary 2, given a formula $\varphi$, we can construct an NBW $N_\varphi$ of size $|{Q_n}|.|{\delta _n}|$ that accepts precisely the computations that satisfy $\varphi$. Thus, $\varphi$ is satisfiable iff $N_\varphi$ is nonempty. In order to prove that the formula is satisfiable we have to show that $N_\varphi$ accepts a word *w* such that some computation $\rho$ satisfies *w*. However, a word *w* is non empty iff every letter  appearing in *w* is non empty. It follows that while testing the non emptiness of $N_\varphi$ we have to follow only transitions using non empty letters in . The nonemptiness of an NBW is tested in nondeterministic logarithmic space. However, as $N_\varphi$ is exponential in $|{\varphi }|$ we get an algorithm working in space polynomial in $|{\varphi }|$. The algorithm constructs $N_\varphi$ on-the-fly. We have to show that the emptiness of letters in  can be tested in space polynomial in $|{\varphi }|$. This follows from Proposition 1 below.

The hardness argument can be proved by a reduction from ltl satisfiability [53]. $\square$

#### Theorem 6

The model-checking problem of ltol is pspace-${\textsc {complete}}$ with respect to $|{Sys}|$ and $|{\varphi }|$.

Note that the stated bounds in terms of $|{Sys}|$ refer to the symbolic representation of the system. The complexity is ${\textsc {logspace}}$ in the size of the corresponding CTS $\mathscr {T}(Sys)$, which is anyway exponentially larger.

#### Proof

Given a finite state system $Sys=\langle {\mathscr {V},\ \rho ,\ \theta }\rangle$ and a set of assertions on state variables $\mathscr {V}$, on ${\textsc {ch}},\ {{\textsc {d}}},\ K$, and on $cv_1,\dots cv_n$. We assume $\rho$ to be total and then we can construct a CTS representation of *Sys* as follows: $\mathscr {T}(Sys)= \langle {\textsc {ch}},\varSigma ,M,S,S_0,R,L,{\textsc {ls}}\rangle$, where the components of $\mathscr {T}(Sys)$ are as follows. $S=\varSigma$ (*L* is the identity function), and thus *S* is the set of possible interpretations of the variables in $\mathscr {V}$, i.e., $S=2^{\mathscr {V}}$. The set of initial states $S_0$ is the set of states *s* such that $s\models \theta$, i.e., $S_0=\{{s\models {\theta }}\}$, and $M={\textsc {ch}}\times 2^{{\textsc {d}}}\times K\times 2^{2^{{\textsc {cv}}}}$. We have that $R(s,m)=\{{s': \left( s,m,s'\right) \models \rho }\}$ and $\emptyset$ otherwise. Furthermore, we consider all states in $\mathscr {T}(Sys)$ to be accepting. The number of states in the transition system $\mathscr {T}(Sys)$ may be exponentially larger than the description of *Sys*. Notice that although *M* is doubly exponential in ${\textsc {cv}}$ the labels of transitions of $\mathscr {T}(Sys)$ are those obtained from  for some *k*. Thus, the number of distinct labels appearing on transitions of $\mathscr {T}(Sys)$ is bounded by $|S|\cdot |{\textsc {ch}}|\cdot 2^{|{\textsc {d}}|}\cdot |K|$.

The system *Sys* satisfies $\varphi$ iff all the computations of *Sys* satisfy $\varphi$, thus for every computation $\rho \in L_{\omega }({\mathscr {T}(Sys)})$ there exists a word $w\in L_{\omega }({N_{\varphi }})$ such that $\rho \models w$. Dually, *Sys* does not satisfy $\varphi$ iff for some computation $\rho \in L_{\omega }({\mathscr {T}(Sys)})$ and for some word $w\in L_{\omega }({N_{\lnot \varphi }})$ we have $\rho \models w$. This is equivalent to check $L_{\omega }({\mathscr {T}(Sys)})\cap \bigcup _{w\in L_{\omega }({N_{\lnot \varphi }})}L_{\omega }({w})=\emptyset$. Since our formulas are in positive normal form, $\lnot \varphi$ can be obtained from $\varphi$ by $\overline{\varphi }$. By Corollary 2, we have that $N_{\lnot \varphi }$ has $2^{\mathscr {O}({|{\varphi }|})}$ states and $|{N_{\lnot \varphi }}|$ is in $2^{\mathscr {O}({|{\varphi }|})}$. Note, however, that the words of $N_{\lnot \varphi }$ are in  while the computations of $\mathscr {T}(Sys)$ are in $(\varSigma \times M)^{\omega }$. The model-checking problem can be reduced to finding a word *w* accepted by $A_{\lnot \varphi }$ and a computation $\rho$ of $\mathscr {T}(Sys)$ such that $\rho \models w$. Recall that $\rho \models w$ if for every $i\ge 0$ we have that $\sigma ^{\rho }_i=\sigma ^{w}_i$ and for every $O\in {\mathsf {obs}({\varphi })}$ we have $m^{\rho }_i\models O$ iff . This amounts to check the nonemptiness problem of a (modified) intersection of $\mathscr {T}(Sys)$ and $N_{\lnot \varphi }$, where the transition $(s,m,s')$ of $\mathscr {T}(Sys)$ can match transitions of $N_{\lnot \varphi }$ that read letters  for . Note that for every $m\in M$ there is a unique  such that . Thus, we check letter by letter that the word *w* accepted by $N_{\lnot \varphi }$ and the computation $\rho$ produced by $\mathscr {T}(Sys)$ are such that $\rho \models w$. Thus, we only need to show that checking  can be tested in space polynomial in $|{\varphi }|$. Indeed, This follows from Proposition 2. Since all states in $\mathscr {T}(Sys)$ are accepting, the construction of $N_{\mathscr {T}(Sys),\lnot \varphi }$ is the product of $\mathscr {T}(Sys)$ with $N_{\lnot \varphi }$ with transitions composed as explained. We have that $N_{\mathscr {T}(Sys),\lnot \varphi }$ has $2^{\mathscr {O}({|{Sys}|+|{\varphi }|})}$ states. Hence, $|{N_{\mathscr {T}(Sys), \lnot \varphi }}|$ is in $2^{\mathscr {O}({|{Sys}|+|{\varphi }|})}$ . We have that $N_{\mathscr {T}(Sys),\lnot \varphi }$ can be constructed on-the-fly and a membership in pspace with respect to $|{Sys}|$ and $|{\varphi }|$, follows from the membership in nlogspace of the nonemptiness problem for NBW. Checking that $Sys\models \varphi$ is in $\mathscr {O}({{|{\varphi }|+|{Sys}|}})$.

The hardness follows from the same hardness results for discrete systems and ltl  [53]. $\square$

The following proposition states that given a letter  we can check whether there exists a message *m* that satisfies  in ${\textsc {np}}$ with respect to $|{{\mathsf {obs}({\varphi })}}|$. Notice that, in particular, $|{{\mathsf {obs}({\varphi })}}|$ should be larger than the number of variables in ${\textsc {cv}}$ and ${\textsc {d}}$ that appear in $\varphi$, the number of agents in *K* that are mentioned in $\varphi$ and the number of channels in ${\textsc {ch}}$ appearing in $\varphi$. Those that do not appear in $\varphi$ can be removed from the message alphabet *M*.

#### Proposition 1

(Observation satisfiability) Consider a letter . Emptiness of  is ${\textsc {np-complete}}$ in $|{{\mathsf {obs}({\varphi })}}|$.

#### Proof

Given a letter  let  be the set of observations in  and the negations of the observations *not* appearing in . That is, . Let  be the conjunction of all observations in . Clearly, the Emptiness of a letter  is equivalent to the satisfiability of . Thus, we can restrict our attention to the satisfaction of an observation. Given an observation *O* let *atom*(*O*) denote the set of subformulas of *O* of the form $\bullet ^{\exists }{O'}$ and $\forall {O'}$.

We show that satisfaction of *O* can be solved in NP as follows:

- select a subset *S* of *atom*(*O*);
- select an assignment to ${\textsc {d}}$, a channel $ch$ and an agent *k*;
- for each $\bullet ^{\exists }{O'}\in S$ guess one assignment to ${\textsc {cv}}$.

Verify that the choice of *S*, the assignment to ${\textsc {d}}$, the channel $ch$ and the agent *k* satisfy *O*. Notice, that the elements of *atom*(*O*) are treated as Boolean values in this check: $O''\in S$ is evaluted as true and $O''\notin S$ is evaluated as false. For each $\bullet ^{\exists }{O'}\in S$ check that the assignment to ${\textsc {cv}}$ guessed for $\bullet ^{\exists }{O'}$ fulfills two conditions:

- the assignment to ${\textsc {cv}}$ together with the assignment to ${\textsc {d}}$, the channel $ch$ and the agent *k* satisfy $O'$.
- For every $\bullet ^{\forall }{O''}\in S$ check that the assignment to ${\textsc {cv}}$ together with the assignment to ${\textsc {d}}$, the channel $ch$ and the agent *k* satisfy $O''$.

Notice that the sum of sizes of the guessed elements is polynomal in the size of *O* and the verification can be completed in polynomial time.

Hardness in ${\textsc {np}}$ follows from the hardness of Boolean satisfiability. $\square$

The following proposition states that checking if a message $m\in M$ satisfies an observation letter  can be tested in ${{\textsc {p}}}^{{\textsc {np}}}$ in $|{{\mathsf {obs}({\varphi })}}|$. We consider the case that $\pi$ is represented as a Boolean formula over ${\textsc {cv}}$. This is reasonable as when considering a transition $(s,m,s')$, where $m=\left( ch,\mathbf{d},i,\pi \right)$, then $\pi$ can be obtained as such a formula from ${g}^{s}_{i}$ by using the values in *s*, $ch$, and $\mathbf{d}$.

#### Proposition 2

(Observation model-checking) Consider a letter  and an observation $m\in M$. Whether  can be tested in ${{\textsc {p}}}^{{\textsc {np}}}$ in $|{{\mathsf {obs}({\varphi })}}|$.

#### Proof

As in the case of Proposition 1 give a letter  we consider . Thus, we restrict our attention to the case of whether *m* satisfies an observation *O*.

Let $m=\left( ch,\mathbf{d},i,\pi \right)$. We simplify *O* by converting every reference to $ch$, ${\textsc {d}}$ or $i$ to the constants appearing in *m*. It follows that we are left with a Boolean combination of $\bullet ^{\exists }{\cdot }$ and $\bullet ^{\forall }{\cdot }$ subformulas, where only variables from ${\textsc {cv}}$ appear.

For a subformula $\bullet ^{\exists }{O'}$ we can check whether $m\models \bullet ^{\exists }{O'}$ by checking whether $\pi \wedge O'$ is satisfiable. For a subformula $\bullet ^{\forall }{O'}$ we can check whether $m\models \bullet ^{\forall }{O'}$ by checking whether $\pi \rightarrow O'$ is valid. Both checks can be accomplished by an ${\textsc {np}}$ oracle.

The problem is ${\textsc {np}}$-hard in $|{{\textsc {cv}}}|$ as $m\models \bullet ^{\exists }{\mathsf{true}}$ holds iff $\pi$ is satisfiable. The problem is co-${\textsc {np}}$-hard in $|{{\textsc {cv}}}|$ as $m\models \bullet ^{\forall }{\mathsf{false}}$ iff $\pi$ is unsatisfiable. We do not know whether the problem is ${{\textsc {p}}}^{{\textsc {np}}}$-complete. $\square$

We note that in the case that *m* is represented as a set of assignments to ${\textsc {cv}}$, we can modify the Boolean value problem [43] to show that  can be evaluated in ${\textsc {logspace}}$.

## Related works

In this section, we present closely related works with respect to (i) traditional formal modelling of Multi-Agent Systems; (ii) distributed and concurrent computation models; (iii) knowledge and strategic formalisms; (iv) business process modelling; and (v) logics for temporal reasoning.

**Traditional formal modelling of MAS** As mentioned before, formal modelling is highly influenced by traditional formalisms used for verification, see [8, 27]. These formalisms are, however, very abstract in that their models representations are very close to their mathematical interpretations (i.e., the underlying transition systems). Although this may make it easy to conduct some logical analysis [9, 22, 50] on models, it does imply that most of the high-level MAS features may only be hard-coded, and thus leading to very detailed models that may not be tractable or efficiently implementable. This concern has been already recognised and thus more formalisms have been proposed, e.g., *Interpreted Systems Programming Language* (ISPL) [42] and MOCHA [7] are proposed as implementation languages of *Interpreted Systems* (IS) [27] and *Reactive Modules* (RM) [8] respectively. They are still either fully synchronous or shared-memory based and thus do not support flexible coordination and/or interaction interfaces. A recent attempt to add dynamicity in this sense has been adopted by *visibly CGS* (vCGS) [14]: an extension of *Concurrent-Game Structures* (CGS) [9] to enable agents to dynamically hide/reveal their internal states to selected agents. However, vCGS relies on an assumption of [11] which requires that agents know the identities of each other. This, however, only works for closed systems with a fixed number of agents.

Other attempts to add dynamicity and reconfiguration include dynamic I/O automata [10], Dynamic reactive modules of Alur and Grosu [6], Dynamic reactive modules of Fisher et al. [30], and open MAS [39]. However, their main interest was in supporting dynamic creation of agents. Thus, the reconfiguration of communication was not their main interest. While ${\textsc {ReCiPe}}$ may be easily extended to support dynamic creation of agents, none of these formalisms may easily be used to control the targets of communication and dissemination of information.

**Distributed and concurrent computation models.** There are a plenty of formalisms that were specifically designed to model concurrent computations and distributed systems, (cf. $\pi$-calculus [48], Psi-calculus [18, 21], *AbC* calculus [2, 5], *Channel Systems* [12], etc). These formalisms rely heavily on message-passing and synchronisation, and except for *AbC* and the broadcast version of Psi-calculus [21] they mostly rely on point-to-point communication mechanisms to establish interaction. Clearly, point-to-point communication is not appropriate to model interaction in MAS settings, and a group-based communication is more appropriate (See [2, 5] for a detailed comparison).

Furthermore, these formalisms also differ in their degree of support to reconfiguration. On one hand, *Channel Systems* cannot deal with reconfiguration and only support fixed communication structures. On the other hand, while $\pi$ and Psi-calculi can support 1-by-1 expansion of the scope of interaction, *AbC* supports a general group communication with sophisticated scoping mechanisms. In ${\textsc {ReCiPe}}$, we extend these ideas to support awareness capabilities, interaction beyond broadcast, and dynamic construction of groups and private group coalitions. It is worth mentioning that although there is a separation result [25] stating that point-to-point and broadcast communication are incomparable, we can still mimic point-to-point communication in ${\textsc {ReCiPe}}$ under closed world assumption. For instance, the team formation protocol in Sect. 6 is such example where robots are recruited one-by-one with a non-deterministic selection. This, of course, works because we assume a closed world settings where no other agents may intervene and disturb such protocol. We conjecture that if ${\textsc {ReCiPe}}$ is extended with mechanisms to allow communicating secret messages, we would be able to encode point-to-point in general.

**Knowledge and Strategic formalisms.** There is a rich literature on modelling and reasoning about knowledge and strategic behaviour in multi-agent systems. They employ techniques to study knowledge dissemination in distributed settings [54, 56] and respectively analysing strategic behaviour [17, 20] to study concepts like Nash equilibria, Pareto optimality and evolutionary strategies.

Reasoning in these settings is known to be hard, and restrictions on the structure of the systems and their communication mechanisms are imposed to mitigate such difficulty. For instance, hierarchical environments [56] , broadcast environments [41], public actions [17], public announcements [54] or gossip spread mechanisms [55]. Most of these approaches rely on an assumption of perfect recall to guarantee the decidability of the verification problem, i.e., all agents of the system are aware of all events that have happened so far. In fact, they keep a complete record of all events they have observed so far and they base their judgement on such complete record.

This assumption can be appropriate in specific distributed applications like Blockchains, supply chains where there is a powerful infrastructure that mediates the interaction and makes global information available to everyone easily. However, this is a strong assumption to be made in general distributed settings. For instance, in applications where a group of robots with limited resources communicate in ad-hoc wireless networks. Also, in applications where security is of concern and only specific agents are allowed to have access to a specific information.

To the best of our knowledge, all proposed broadcast settings in the context of knowledge and strategic reasoning imply more than a broadcast in actual communication. That is, in broadcast environments [41] and public actions [17] the communication is totally deterministic and all agents have access to all events that have happened so far and their respective order. This, of course, simplifies the verification problem because it removes all possible sources of information forks (the source of undecidability for some verification tasks). That is, an agent who is not targeted by the communication still knows the events that have happened. In normal broadcast like in ${\textsc {ReCiPe}}$, non-targeted agents are unaware of the communication and cannot have access to communications that they did not participate in. In fact, using a perfect recall gives the power to agents to even count how many communication steps have happened so far. This is, clearly, unreasonable in distributed settings because one agent might participate for a finite time in the communication protocol and stays idle most of the time. Allowing agents to have access to that much of information does not come without a cost, it requires memory and, although decidable, it is not surprising that the complexity of verification in such settings is very high.

Other than the fact that all of the mentioned approaches rely on flat or static communication structure, the main difference with respect to our way of dissemination of information in ${\textsc {ReCiPe}}$ is due to the fact that we are handling a different problem, i.e., a coordination problem. In fact, a coordination problem is a subproblem of general knowledge dissemination. The classic examples of the latter assume a very powerful communication infrastructure and usually ask whether a group of uninformed agents can become totally informed about the state of the whole system after a number of communication rounds. In that settings, an event is made publicly accessible to all agents after being executed, and thus each agent is able to keep track of the state of the system during execution. Although that agent may not know the exact exchange of information, it can still make deductions based on the history of communications. The logic of gossiping [55] gives an excellent characterisation of the different cases of communications in point-to-point settings. From our perspective, we consider the asynchronous case to be the more representative case to coordination in distributed settings as agents only observe the communication they participate in. In a coordination problem, the objective is different as agents can be arbitrarily informed about the state of the system. Some agents might be totally uninformed and stay so because they do not interact with the rest of the systems while other agents might need to know some more information at some point during execution and then forget about it later. Thus, we believe that in a dynamic and reconfigurable communication structure a perfect recall is not required, given that communication can be introduced on need-basis to resolve information forks.

**Business process modelling** Some approaches from the realm of busniess process modelling such as Artifact-centric systems [16] have some similarities with respect to ${\textsc {ReCiPe}}$. An artifact system consists of two main components: *data* which models the local state alongside the view of the agent on the environment; and *lifecycle* which models the behaviour of agents working on the data. Artifact-centric systems define agents’ local states as evolving database instances and can be considered as a specialisation of interpreted systems were local states are structured in form of databases. Agents interact among themselves and with an environment comprising all artifacts in the system. That is, all local states of agents are considered as partial views of a holistic environment database. Thus, the environment can be represented as a special agent holding the combined knowledge of all agents in the system. There are two main differences with respect to ${\textsc {ReCiPe}}$: (1) there is no notion of reconfiguration of interaction interfaces in artifact systems and all agents have to participate in every interaction even to just stay idle; (2) considering the environment as a special agent composed with the rest of the system restricts its capabilities. That is, the environment can no longer be able to simultaneously trigger multiple events with respect to different agents local views. In other words, this restriction reduces the power of the environment and forces it to interleave and alternate behaviour with respect to other agents. In ${\textsc {ReCiPe}}$, the environment can be more powerful because some local variables of agents may abstract incomparable local views of the environment. Changes in the assignments to these variables cannot be restricted, and thus the environment can change them simultaneously resulting in triggering multiple events for the different agents which have to coordinate their executions.

**Logics for temporal reasoning** As for logics we differ from traditional languages like ltl and ctl in that our formula may refer to messages and their constraints. This is, however, different from the atomic labels of pdl [29] and modal $\mu$-calculus [40] in that ltol mounts complex and structured observations on which designers may predicate on. Thus the interpretation of a formula includes information about the causes of variable assignments and the interaction protocols among agents. Such extra information may prove useful in developing compositional verification techniques.

## Concluding remarks

We introduced a formalism that combines message-passing and shared-memory to facilitate realistic modelling of distributed multi agent systems. A system is defined as a set of distributed agents that execute concurrently and only interact by message-passing. Each agent controls its local behaviour as in Reactive Modules [8, 30] while interacting externally by message passing as in $\pi$-calculus-like formalisms [2, 5, 48]. Thus, we decouple the individual behaviour of an agent from its external interactions to facilitate reasoning about either one separately. We also make it easy to model interaction features of MAS, that may only be tediously hard-coded in existing formalisms.

We introduced an extension to ltl, named ltol, that characterises messages and their targets. This way we may not only be able to reason about the intentions of agents in communication, but also we may explicitly specify their interaction protocols. Finally, we provided a novel automata construction that permits satisfiability and model-checking in space polynomial with respect to the size of the formula and the size of the system. This is a major improvement on the early results in [1] that were in expspace with respect to the number of common variables and pspace-complete with respect to the rest of the input.

**Future works** We plan to provide tool support for ${\textsc {ReCiPe}}$, but with a more user-friendly syntax. We would like to provide a light-weight programming-language-like syntax to further simplify modelling.

We want to exploit the interaction mechanisms in ${\textsc {ReCiPe}}$ and the extra information in ltol formulas to conduct verification compositionally. As mentioned, we believe that relating to sender intentions will facilitate that.

We intend to study the relation with respect to temporal epistemic logic [35]. Although we do not provide explicit knowledge operators, the combination of data exchange, receivers selection, and enabling/disabling of synchronisation based on the evolving states of the different agents, allow them to dynamically deduce information about each other. Furthermore we want to study ${\textsc {ReCiPe}}$ under dynamic creation of agents while reconfiguring communication. Thanks to the new compositional semantics in terms of CTS, the dynamic creation of agents can now be easily linked to the execution of some blocking transitions. To give an intuition about a (possible) extension consider the following semantic rule:

2 $$\begin{aligned} {A_1 \xrightarrow {(\upsilon ,?,c)}A'_1\Vert A_1}{} \end{aligned}$$

Here, we use $A\xrightarrow {m}A'$ to denote that in state *s* agent *A* may receive a message *m* and evolves to $A'$ with state $s'$, i.e., $(s,m,s')\in R_A$. Clearly, the semantic rule 2 indicates that the agent replicates itself once a multicast message on *c* is received as a side-effect of interaction. Thus, if we compose $A_1$ with some other agent, say $A_2$, such that $A_2\xrightarrow {(\upsilon ,!,c)}A'_2$, the following transition is derivable by the semantics of parallel composition in Definition 3:

3 $$\begin{aligned} \frac{\text {if}\quad A_1\xrightarrow {(\upsilon ,?,c)}A'_1\Vert A_1\qquad \text {and}\qquad A_2\xrightarrow {(\upsilon ,!,c)}A'_2}{A_1\Vert A_2 \xrightarrow {(\upsilon ,!,c)}A'_1\Vert A_1\Vert A_2} \end{aligned}$$

Namely, a new replica of $A_1$ is dynamically created when agents exchanged a specific message. Agent $A_1$ can be thought of as a server that spawn a new thread to handle concurrent requests from clients.

Finally, we want to target the distributed synthesis problem [28]. Several fragments of the problem have been proven to be decidable, e.g., when the information of agents is arranged hierarchically [19], the number of agents is limited [34], or the actions are made public [15]. We conjecture that the ability to disseminate information and reason about it might prove useful in this setting.

## A Proofs for Section 4 (Channelled Transition Systems)

### Lemma 1

(Commutativity and Associativity) Given two CTS $\mathscr {T}_1$ and $\mathscr {T}_2$ we have that:

- $$\Vert $$ is commutative: $$\mathscr {T}_1\Vert \mathscr {T}_2=\mathscr {T}_2\Vert \mathscr {T}_1$$;
- $$\Vert $$ is associative: $$(\mathscr {T}_1\Vert \mathscr {T}_2) \Vert \mathscr {T}_3=\mathscr {T}_1\Vert (\mathscr {T}_2 \Vert \mathscr {T}_3)$$.

Note that Lemma 1 is crucial to ensure that our parallel compostion operator is a commutative monoid, as otherwise it would not represent the right behaviour of interacting programs.

### Proof

We prove each statement separately. In both statement, the proof proceeds by case analysis on the joint transition.

- **(**$\Vert$ **is commutative)**: we consider all possible joint transitions from $\mathscr {T}_1\Vert \mathscr {T}_2$ and we show that they have corresponding transitions in $\mathscr {T}_2\Vert \mathscr {T}_1$ and vice versa. We only show one direction and the other direction follows in a similar way.
  - **Case** $\left( \upsilon ,!,c\right)$: By Definition 3, we have that $((s_1,s_2),\left( \upsilon ,!,c\right) , (s'_1,s'_2))\in R_{12}$ ($R_{12}$ is the transition relation of $\mathscr {T}_1\Vert \mathscr {T}_2$) in the following cases:
    1. $(s_1,\left( \upsilon ,!,c\right) ,s'_1)\in R_1$ and $(s_2,\left( \upsilon ,?,c\right) ,s'_2)\in R_2$: It follows that $((s_2,s_1),\left( \upsilon ,!,c\right) , (s'_2,s'_1))\in R_{21}$ ($R_{21}$ is the relation of $\mathscr {T}_2\Vert \mathscr {T}_1$) is derivable because $(s_2,\left( \upsilon ,?,c\right) ,s'_2)\in R_2$ and $(s_1,\left( \upsilon ,!,c\right) ,s'_1)\in R_1$;
    2. $(s_1,\left( \upsilon ,!,c\right) ,s'_1)\in R_1$ and $c\notin {\textsc {ls}}^{2}(s_2)$ and ($s_2=s'_2$): It follows that $((s_2,s_1),\left( \upsilon ,!,c\right) , (s'_2,s'_1))\in R_{21}$ is derivable because $c\notin {\textsc {ls}}^{2}(s_2)$ and ($s_2=s'_2$) and $(s_1,\left( \upsilon ,!,c\right) ,s'_1)\in R_1$;
    3. $(s_1,\left( \upsilon ,!,\star \right) ,s'_1)\in R_1$ and ($s_2=s'_2$) and $\forall s''_2. (s_2,\left( \upsilon ,?,c\right) ,s''_2)\notin R_2$: It follows that $((s_2,s_1),\left( \upsilon ,!,\star \right) , (s'_2,s'_1))\in R_{21}$ is derivable because ($s_2=s'_2$) and $\forall s''_2. (s_2,\left( \upsilon ,?,c\right) ,s'_2)\notin R_2$ and $(s_1,\left( \upsilon ,!,c\right) ,s'_1)\in R_1$;
    4. $(s_2,\left( \upsilon ,!,c\right) ,s'_2)\in R_2$ and $(s_1,\left( \upsilon ,?,c\right) ,s'_1)\in R_1$: This is the symmetric case of case (1);
    5. $(s_2,\left( \upsilon ,!,c\right) ,s'_2)\in R_2$ and $c\notin {\textsc {ls}}^{1}(s_1)$ and ($s_1=s'_1$): This is the symmetric case of case (2);
    6. $(s_2,\left( \upsilon ,!,\star \right) ,s'_2)\in R_2$ and ($s_1=s'_1$) and $\forall s''_1. (s_1,\left( \upsilon ,?,c\right) ,s''_1)\notin R_1$: This is the symmetric case of case (3).
  - **Case** $\left( \upsilon ,?,c\right)$**:** By Definition 3, we have that $((s_1,s_2),\left( \upsilon ,?,c\right) , (s'_1,s'_2))\in R_{12}$ in the following cases:
    1. $(s_1,\left( \upsilon ,?,c\right) ,s'_1)\in R_1$ and $(s_2,\left( \upsilon ,?,c\right) ,s'_2)\in R_2$: It follows that $((s_2,s_1),\left( \upsilon ,?,c\right) , (s'_2,s'_1))\in R_{21}$ is derivable for the same reason;
    2. $(s_1,\left( \upsilon ,?,c\right) ,s'_1)\in R_1$ and $c\notin {\textsc {ls}}^{2}(s_2)$ and ($s_2=s'_2$): It follows that $((s_2,s_1),\left( \upsilon ,?,c\right) , (s'_2,s'_1))\in R_{21}$ is derivable because $c\notin {\textsc {ls}}^{2}(s_2)$ and ($s_2=s'_2$) and $(s_1,\left( \upsilon ,?,c\right) ,s'_1)\in R_1$;
    3. $(s_2,\left( \upsilon ,?,c\right) ,s'_2)\in R_2$ and $c\notin {\textsc {ls}}^{1}(s_1)$ and ($s_1=s'_1$): This is the symmetric case of case (2);
    4. $(s_1,\left( \upsilon ,?,\star \right) ,s'_1)\in R_1$ and ($s_2=s'_2$) and $\forall s''_2. (s_2,\left( \upsilon ,?,c\right) ,s''_2)\notin R_2$: It follows that $((s_2,s_1),\left( \upsilon ,?,\star \right) , (s'_2,s'_1))\in R_{21}$ is derivable because ($s_2=s'_2$) and $\forall s''_2. (s_2,\left( \upsilon ,?,c\right) ,s''_2)\notin R_2$ and $(s_1,\left( \upsilon ,?,c\right) ,s'_1)\in R_1$;
    5. $(s_2,\left( \upsilon ,?,\star \right) ,s'_2)\in R_2$ and ($s_1=s'_1$) and $\forall s''_1. (s_1,\left( \upsilon ,?,c\right) ,s''_1)\notin R_1$: This is the symmetric case of case (3).
- **(**$\Vert$ **is associative):** we consider all possible joint transitions from $(\mathscr {T}_1\Vert \mathscr {T}_2) \Vert \mathscr {T}_3$ and we show that they have corresponding transitions in $\mathscr {T}_1\Vert (\mathscr {T}_2 \Vert \mathscr {T}_3)$ and vice versa. We only show one direction and the other direction follows in a similar way.
  - **Case** $\left( \upsilon ,!,c\right)$**:** By Definition 3, we have $(((s_1,s_2),s_3),\left( \upsilon ,!,c\right) , ((s'_1,s'_2),s'_3))\in R_{(12)3}$ in the following cases:
    1. $((s_1,s_2),\left( \upsilon ,!,c\right) ,(s'_1,s'_2))\in R_{12}$ and $(s_3,\left( \upsilon ,?,c\right) ,s'_3)\in R_3$: As before, there are six cases for $((s_1,s_2),\left( \upsilon ,!,c\right) ,(s'_1,s'_2))\in R_{12}$, we only consider the case ($(s_1,\left( \upsilon ,!,c\right) ,s'_1)\in R_1$ and $(s_2,\left( \upsilon ,?,c\right) ,s'_2)\in R_2$); and other cases follow similarly. It follows that $((s_1,(s_2,s_3)),$ $\left( \upsilon ,!,c\right) , (s'_1,(s'_2,s'_3))\in R_{1(23)}$ where $(s_1,\left( \upsilon ,!,c\right) ,s'_1)\in R_1$ and
      $((s_2,s_3),\left( \upsilon ,?,c\right) , (s'_2,s'_3))\in R_{23}$ such that $(s_2,\left( \upsilon ,?,c\right) , s'_2)\in R_2$ and $(s_3,\left( \upsilon ,?,c\right) , s'_3)\in R_3$ as required;
    2. $((s_1,s_2),\left( \upsilon ,!,c\right) ,(s'_1,s'_2))\in R_{12}$ and $c\notin {\textsc {ls}}^{3}(s_3)$ and ($s_3=s'_3$): It follows that $((s_1,(s_2,s_3)),\left( \upsilon ,!,c\right) , (s'_1,(s'_2,s'_3))\in R_{1(23)}$ where $(s_1,\left( \upsilon ,!,c\right) ,s'_1)\in R_1$ and $((s_2,s_3),\left( \upsilon ,?,c\right) , (s'_2,s'_3))\in R_{23}$ such that $(s_2,\left( \upsilon ,?,c\right) , s'_2)\in R_2$ and $c\notin {\textsc {ls}}^{3}(s_3)$ and ($s_3=s'_3$);
    3. $((s_1,s_2),\left( \upsilon ,!,\star \right) ,(s'_1,s'_2))\in R_{12}$ and ($s_3=s'_3$) and $\forall s''_3. (s_3,\left( \upsilon ,?,\star \right) ,$ $s'_3)\notin R_3$: It follows that $((s_1,(s_2,s_3)),\left( \upsilon ,!,\star \right) , (s'_1,(s'_2,s'_3))\in R_{1(23)}$ where $(s_1,\left( \upsilon ,!,\star \right) ,s'_1)\in R_1$ and $((s_2,s_3),\left( \upsilon ,?,\star \right) , (s'_2,s'_3))\in R_{23}$ such that $(s_2,\left( \upsilon ,?,\star \right) , s'_2)\in R_2$ and ($s_3=s'_3$) and $\forall s''_3. (s_3,\left( \upsilon ,?,\star \right) ,$ $s'_3)\notin R_3$;
    4. $((s_1,s_2),\left( \upsilon ,?,c\right) ,(s'_1,s'_2))\in R_{12}$ and $(s_3,\left( \upsilon ,!,c\right) ,s'_3)\in R_3$: This is the symmetric case of case (1);
    5. $(s_3,\left( \upsilon ,!,c\right) ,s'_3)\in R_3$ and $c\notin {\textsc {ls}}^{12}(s_1,s_2)$ and ($(s_1,s_2)=(s'_1,s'_2)$): This is the symmetric case of case (2);
    6. $$(s_3,\left( \upsilon ,!,\star \right) ,s'_3)\in R_3$$ and $$(s_1,s_2)=(s'_1,s'_2)$$ and $$\forall (s''_1,s''_2).$$
      $((s_1,s_2),\left( \upsilon ,?,c\right) ,(s''_1,s''_2))\notin R_1$: This is symmetric to case (3).
  - **Case** $\left( \upsilon ,?,c\right)$**:** it follows similarly by case analysis on Definition 3.

$$\square $$

### Lemma 2

(Non-blocking Broadcast) Given a CTS $\mathscr {T}_1$ and for every other CTS $\mathscr {T}$, we have that for every reachable state $(s_1,s)$ of $\mathscr {T}_1\Vert \mathscr {T}$ the following holds.

$$\begin{aligned} (s_1,(\upsilon ,!,\star ),s'_1)\in R_1\ \hbox {implies}\ ((s_1,s),(\upsilon ,!,\star ),(s'_1,s'))\in R_{\mathscr {T}_1\Vert \mathscr {T}} \end{aligned}$$

### Proof

By Definition 3, we have only two cases to derive $((s_1,s),(\upsilon ,!,\star ),(s'_1,s'))\in R_{\mathscr {T}_1\Vert \mathscr {T}}$ given that $(s_1,(\upsilon ,!,\star ),s'_1)\in R_1$. Note that, by definition, the condition $\star \in {\textsc {ls}}^{k}({s})$ always holds for any agent *k* and in any state *s*. We show that when the channel is a broadcast $\star$, the receiver does not play any role in enabling the transmission on the channel. In other words, it is only sufficient to have a sender to enable a broadcast at system level. More precisely, if $(s_1,(\upsilon ,!,\star ),s'_1)\in R_1$ then we have the following:

- $$((s_1,s),(\upsilon ,!,\star ),(s'_1,s'))\in R_{\mathscr {T}_1\Vert \mathscr {T}}$$ because $$(s,(\upsilon ,?,\star ),s')\in R$$; or
- $$((s_1,s),(\upsilon ,!,\star ),(s'_1,s'))\in R_{\mathscr {T}_1\Vert \mathscr {T}}$$ because ($$s=s'$$) and $$\forall s''. (s,\left( \upsilon ,?,\star \right) ,s')\notin R_3$$.

Namely, whether there exists a receiver or not, a broadcast can always happen (cannot be blocked). $\square$

### Lemma 3

(Blocking Multicast) Given a CTS $\mathscr {T}_1$ and a multicast channel $c\in C\backslash \{{\star }\}$ such that $(s_1,(\upsilon ,!,c),s'_1)\in R_1$, then for every other CTS $\mathscr {T}$ we have that in every reachable state $(s_1,s)$ of $\mathscr {T}_1\Vert \mathscr {T}$ the following holds.

### Proof

We show that it is not sufficient to only have a sender on a multicast channel *c* to enable a send transition at system level. We require that all listening/connected agents to that channel being able to jointly receive the transmitted message. This also implies that if no one is listening then the transition can happen. By Definition 3, there exist only two cases where $((s_1,s),(\upsilon ,!,c),(s'_1,s'))\in R_{\mathscr {T}_1\Vert \mathscr {T}}$ given that $(s_1,(\upsilon ,!,c),s'_1)\in R_1$. More precisely, if $(s_1,(\upsilon ,!,c),s'_1)\in R_1$ then we have the following:

- $((s_1,s),(\upsilon ,!,c),(s'_1,s'))\in R_{\mathscr {T}_1\Vert \mathscr {T}}$ because $(s,(\upsilon ,?,c),s')\in R$. This implies that $c\in {\textsc {ls}}(s)$; or
- $$((s_1,s),(\upsilon ,!,c),(s'_1,s'))\in R_{\mathscr {T}_1\Vert \mathscr {T}}$$ because ($$s=s'$$) and $$c\notin {\textsc {ls}}(s)$$.

Namely, either all receivers jointly participate or no one is listening, as otherwise the multicast on *c* is blocked. The other direction of the proof follows similarly. $\square$
